# Evolution and impact of bias in human and machine learning algorithm interaction

**DOI:** 10.1371/journal.pone.0235502

**Published:** 2020-08-13

**Authors:** Wenlong Sun, Olfa Nasraoui, Patrick Shafto

**Affiliations:** 1 Department of Computer Engineering and Computer Science, University of Louisville, Louisville, Kentucky, United States of America; 2 Department of Mathematics and Computer Science, Rutgers University - Newark, Newark, New Jersey, United States of America; Shandong University of Science and Technology, CHINA

## Abstract

Traditionally, machine learning algorithms relied on reliable labels from experts to build predictions. More recently however, algorithms have been receiving data from the general population in the form of labeling, annotations, etc. The result is that algorithms are subject to bias that is born from ingesting unchecked information, such as biased samples and biased labels. Furthermore, people and algorithms are increasingly engaged in interactive processes wherein neither the human nor the algorithms receive unbiased data. Algorithms can also make biased predictions, leading to what is now known as algorithmic bias. On the other hand, human’s reaction to the output of machine learning methods with algorithmic bias worsen the situations by making decision based on biased information, which will probably be consumed by algorithms later. Some recent research has focused on the ethical and moral implication of machine learning algorithmic bias on society. However, most research has so far treated algorithmic bias as a static factor, which fails to capture the dynamic and iterative properties of bias. We argue that algorithmic bias interacts with humans in an iterative manner, which has a long-term effect on algorithms’ performance. For this purpose, we present an iterated-learning framework that is inspired from human language evolution to study the interaction between machine learning algorithms and humans. Our goal is to study two sources of bias that interact: the process by which people select information to label (human action); and the process by which an algorithm selects the subset of information to present to people (iterated algorithmic bias mode). We investigate three forms of iterated algorithmic bias (personalization filter, active learning, and random) and how they affect the performance of machine learning algorithms by formulating research questions about the impact of each type of bias. Based on statistical analyses of the results of several controlled experiments, we found that the three different iterated bias modes, as well as initial training data class imbalance and human action, do affect the models learned by machine learning algorithms. We also found that iterated filter bias, which is prominent in personalized user interfaces, can lead to more inequality in estimated relevance and to a limited human ability to discover relevant data. Our findings indicate that the relevance blind spot (items from the testing set whose predicted relevance probability is less than 0.5 and who thus risk being hidden from humans) amounted to 4% of all relevant items when using a content-based filter that predicts relevant items. A similar simulation using a real-life rating data set found that the same filter resulted in a blind spot size of 75% of the relevant testing set.

## Introduction

Websites and online services offer large amounts of information, products, and choices. This information is only useful to the extent that people can find what they are interested in. All existing approaches aid people by suppressing information that is determined to be disliked or not relevant. Thus, all of these methods, by gating access to information, have potentially profound implications for what information people can and cannot find, and thus what they see, purchase, and learn. There are two major adaptive paradigms to help sift through information: information retrieval and recommender systems. Information retrieval techniques [1–9] have given rise to the modern search engines which return relevant results, following a user’s explicit query. For instance, in the probabilistic retrieval model [2], optimal retrieval is obtained when search results are ranked according to their relevance probabilities. Recommender systems, on the other hand, generally do not await an explicit query to provide results [10–17]. Recommender systems can be divided based on which data they use and how they predict user ratings. The first type is content-based filtering (CBF) algorithms [12, 18–24]. It relies on item attributes or user demographics, but often not relations between users (i.e. social relations), as data. Collaborative Filtering (CF) [10, 17, 25–30], on the other hand, does not require item attributes or user attributes. Rather it makes predictions about what a user would like based on what other similar users liked. Both adopt algorithms, e.g. K-nearest neighbors [31, 32] and non-negative matrix factorization (NMF) [33–37], that have close analogs in the psychology literatures on concept learning, e.g. exemplar models [38–40] and probabilistic topic models [41, 42].

Information filtering algorithms [11, 43–45] similarly provide users with a list of relevant results, but do so in response to a query. One classic example is the Rocchio filter [46–48], which modifies the user’s initial query after a first iteration of search to help filter less relevant results. The query is modified based on the set of initial search result documents which are labeled by the user as relevant and non-relevant, respectively. The new query (which is treated like a pseudo-document) is modified by adding and subtracting a weighted combination of relevant and non-relevant documents, respectively. This is quite similar to content-based recommendation, where information about the items is used to rank potentially relevant results.

Common to both recommender systems and information filters is: (1) selection, of a subset of data about which people express their preference, by a process that is not random sampling, and (2) an iterative learning process in which people’s responses to the selected subset are used to train the algorithm for subsequent iterations. The data used to train and optimize performance of these systems are based on human actions. Thus, data that are observed and omitted are not randomly selected, but are the consequences of people’s choices. Recommendation systems suggest items predicted to be of interest to a user (e.g. movies, books, news) based on their user profile [12, 13, 26]. The prediction can be based on people’s explicit (e.g. ratings) or implicit (e.g. their browsing or purchase history) data [49–54], or even query patterns [55]. Research into human choice suggests that both explicit and implicit choices systematically vary based on context, especially the other options that are present when choosing [56–59].

In addition to the simple effects of the interaction between algorithms’ recommendations and people’s choices, people may reason about the processes that underlie the algorithms. Research in cognitive science has shown that people reason about evidence selected by other people. In [60], a computational framework was proposed for modeling how people’s inferences may change as a consequence of reasoning about why data were selected. This framework has been formalized in learning from helpful and knowledgeable teachers [61–64], deceptive informants [65], and epistemic trust [66–68]. People’s reasoning about the intentional nature of the algorithms may exacerbate the effects of cyclic interaction between the algorithms’ recommendations and people’s choices.

We propose a framework for investigating the implications of interactions between human and algorithms, that draws on diverse literature to provide algorithmic, mathematical, computational, and behavioral tools for investigating human-algorithm interaction. Our approach draws on foundational algorithms for selecting and filtering of data from computer science, while also adapting mathematical methods from the study of cultural evolution [69–71] to formalize the implications of iterative interactions.

In our approach, we focus on the primary sources and consequences of bias in collected data. The primary sources of bias come from algorithms and humans. Algorithms, such as recommender systems, filter information in order to present humans with preferred content. After receiving more labels from humans, machine learning algorithms are trained to predict future recommendations. Unlike standard learning theory, the training data are no longer randomly sampled. This in return puts in questions the guarantees about learning from such data. The second source of bias comes from humans. In addition to receiving iterated information optimized to their preferences, humans are not required to provide labels for the presented data. Humans’ choices, when labelling data, are also highly non-random, and would reflect not only their opinions about the presented content, but also inferences about why the content was presented. Finally, the bias that is introduced into the data at any point can be amplified after retraining models and this in turn would further impact the predictions or recommendations. Either of the individual sources of bias could yield instability, thus motivating the need for a better understanding of the performance of such systems in the context of human-algorithm interactions. Specifically, there is a need for a better understanding of the conditions under which we can expect the emergence of systematic bias in the selection of information by algorithms, and for the identification of the conditions under which we can “undo” the effects of these biases to obtain accurate estimates from biased data. We expect the findings to contribute insights back to the fundamental psychology of human reasoning, choice and learning and to the fundamental computer science of learning, recommendation and information filtering.

## Related work

### Iterated learning and language evolution

**Fig 1:**
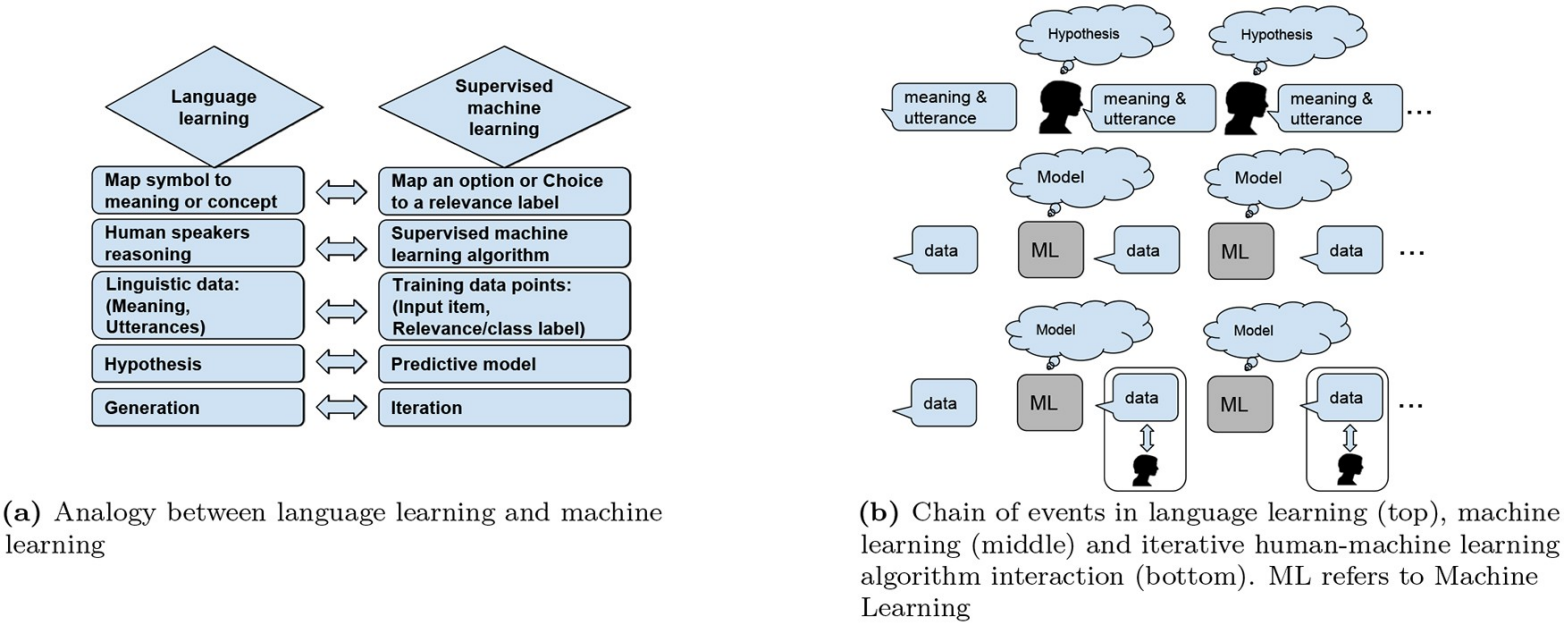
Language learning vs. machine learning. Language learning is analogous to machine learning in several aspects, such as ‘hypothesis’ to ‘model’ in sub-figure (a). Learning a language which gets transmitted throughout consecutive generations of human speakers is analogous to learning a model through consecutive iterations of online machine learning in sub-figure (b). In the iterative human-machine learning algorithm interaction, the output from ML affects human behavior and human also interact with the output which affects next iteration.

In language learning, humans form their own mapping rules after listening to others, and then speak the language following the rules they learned, which will affect the next learner. Language learning and machine learning have several properties in common (see Fig 1). For example, a ‘hypothesis’ in language is analogous to a ‘model’ in machine learning. Learning a language which gets transmitted throughout consecutive generations of humans is analogous to learning an online model throughout consecutive iterations of machine learning [69].

More specifically, we point to the work of Griffith [69] because it was the first work that showed formally, through a Markov chain mathematical framework how the iterated learning within a language learning scenario converges to the prior in the absence of a dependence between the previous iteration’s output and the next iteration’s input.

Although the work of Griffith is situated within the context of language evolution, or more specifically in the context of human learning of language throughout several generations, it is “analogous”, in the sense of information flow and adaptation (and not the actual application domain), to the problem of supervised machine learning, where a model (instead of language) is learned iteratively throughout several iterations (instead of generations).

Thus we consider language evolution more as an “analogous”, rather than a “similar” setting to the iterated machine learning in this paper. We further extend the derivations from Griffith et al. [69] derivations which have been done within a language learning framework, to the realistic framework of dependent interactions between a model and a human in an iterated learning setting, which is the setting for this paper. More specifically in our setting, there is a dependency between the previous output and the next input, and it is this dependency that can generate iterated bias.

**Fig 2:**
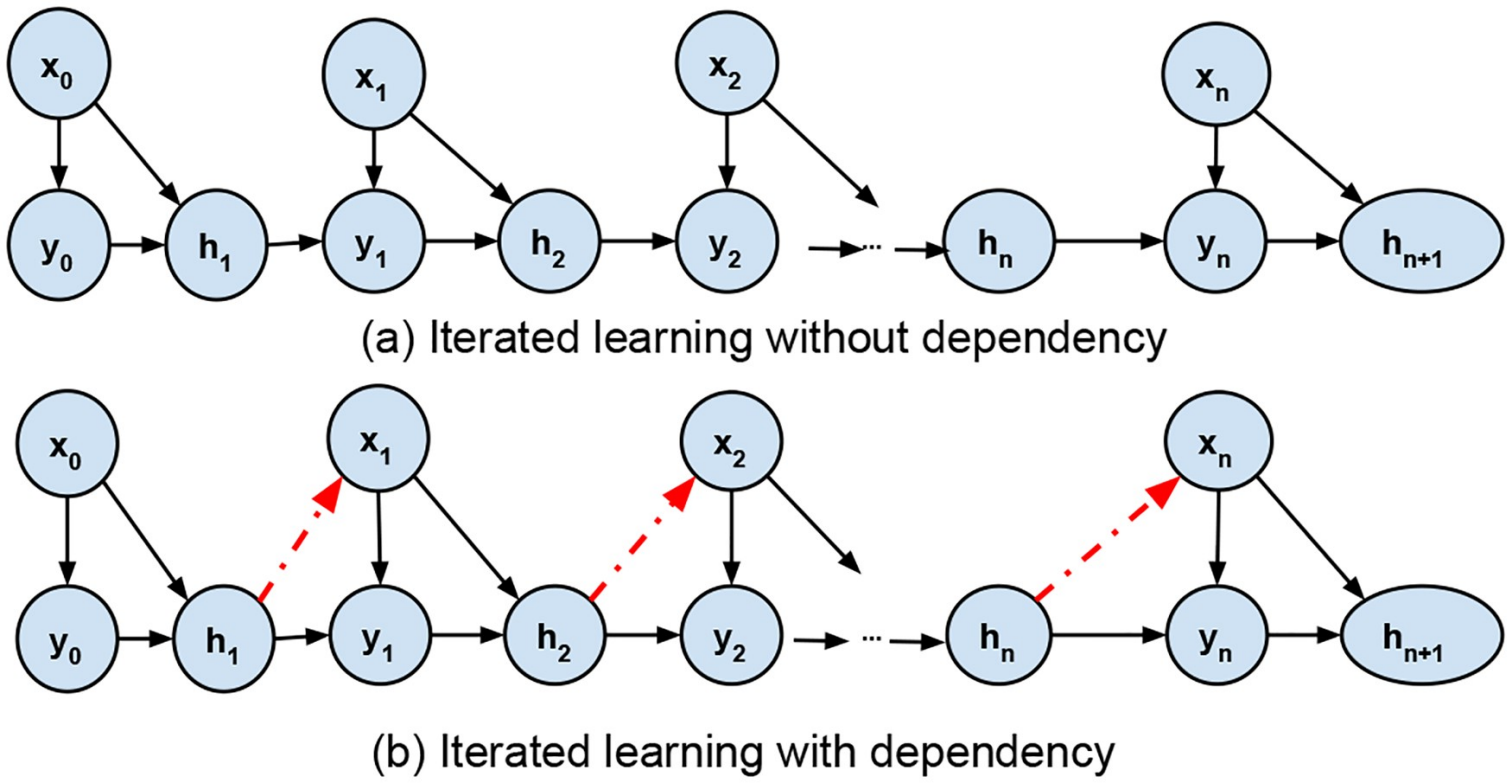
Markov chains for iterated learning with (bottom) and without (top) dependency from previous iterations.

Iterated learning was found to produce meaningful structure patterns in language learning [72–76]. Language evolution can be modeled as a Markov chain, as shown in Fig 2(A). The first learner is exposed to linguistic data and forms an initial hypothesis, before producing their own data, that will serve as the input to the next learner. After sufficient iterations, this process generates a certain hypothesis. This iterated learning chain is expected to converge the hypothesis to the prior distribution of all hypotheses in case that the learner is a Bayesian learner [69]. What this means is that the knowledge learned is not accumulated during the whole process. We refer to this iterated learning model as *pure iterated learning (PIL)*. One problem about this iterated language learning model is the difficulty to prove the convergence or the boundary practically, even if it is proved theoretically [74]. Rafferty et al. gave an upper bound for the convergence, which is *nlog*(*n*) iterations for Bayesian learning of the ranking of *n* constraints or the *n* binary parameters values [77].

Perfors et al. showed that, when certain assumptions about the independence of language and the world are abandoned, the learners converge to languages that depend on the structure as well as their prior biases [78]. In Fig 2(A), there is no dependency between current input *x* and the previous learned hypothesis, which represents the graphic model of PIL. While in Fig 2(B), current input data *x* has dependency on the previous hypothesis, which in the modern world is more realistic.

While iterated learning ensembles the online learning process [79], they differ in several ways. Online learning occurs in a consecutive manner, with the learner providing an answer to a given question in each round. The learner answers the questions using a prediction model or hypothesis, that maps from sets of questions to the set of answers. After prediction, the quality is measured based on the true answers. The goal is to minimize the cumulative loss in the online learning process [80]. Meanwhile, in iterated learning, we are interested in investigating the given information’s effect on the learned hypothesis.

### Relationship between iterated learning and information retrieval

It is interesting to recognize how Iterated Learning manifests itself in the context of adaptive information filters, as exemplified by modern search engines. Based on information retrieval, modern search engines return relevant results, following a user’s explicit query [7]. For instance, in the probabilistic retrieval model, optimal retrieval is obtained when search results are ranked according to their relevance probabilities [2]. Recommender systems, on the other hand, generally do not await an explicit query to provide results [16]. Both Information retrieval and recommender system, to some extent, require information selection to get better results, thus iterated interactive learning naturally fits the purpose of studying the interaction of algorithms and humans.

### Relationship between iterated algorithmic bias and other types of bias

In statistics, bias refers to the systematic distortion of a statistic. Here we can distinguish a biased sample, which means a sample that is incorrectly assumed to be a random sample of a population, and estimator bias, which results from an estimator whose expectation differs from the true value of the parameter [81]. Within our scope, bias is closer to the sample bias and estimator bias from statistics; however, we are interested in what we call **‘iterated algorithmic bias‘** which is the dynamic bias that occurs during the selection by machine learning algorithms of data to show to the user to request labels in order to construct more training data, and subsequently update their prediction model, and how this bias affects the learned (or estimated) model in successive iterations.

Algorithmic bias can be categorized based on the time in which it occurs during the machine learning process [95]. Generally, selecting biased training samples leads to a biased model [96]. Training data bias may come from various sources which depend on the application, such as human labeling, sample selection and others. Several machine learning techniques have been proposed to deal with this problem [87–94]. The post-algorithmic bias emerges when users interpret the output of machine learning algorithms [97–100]. Table 1 lists several common biases and compares them with our iterated algorithmic bias based on several properties.

**Table 1:** Different bias types studied in recent research. Iterated algorithmic bias happens when an algorithm interacts with human response continuously, and updates its model after receiving feedback from the human. Meanwhile, the algorithm interacts with the human by showing only selected items or options. Other types of bias are static, which means they have a one-time influence on an algorithm.

Bias type	Iterative	Ethical issue	Pre-algorithm	Post-algorithm	Research
Feature	✘	✓	✓	✘	[82–86]
Human label	✘	✓	✓	✘	[87–90]
Sample selection	✘	✓	✓	✘	[91–94]
**Iterated algorithmic**	✓	✓	✓	✓	this paper

Recent research pointed to the need to pay attention to bias and fairness in machine learning [101–105]. Some research has studied different forms of biases, some are due to the algorithms while others are due to inherent biases in the input data or in the interaction between data and algorithms [85, 97, 106–113]. Some research on algorithmic bias has focused on the ethical problems that machine learning algorithms might create [82–86, 114–116]. For instance, Zook et al. [82] have recently argued that researchers must carefully check the impact of algorithms on specific groups of people (such as defined by gender and race) before deploying algorithms. Kirkpatrick [83] illustrated the ethical problems that can occur when algorithmic bias is introduced in the justice system. Garcia [85] stated that algorithmic bias may worsen racist bias in certain circumstances. More recently, Kate Crawford’s presentation related to research on the fairness of machine learning algorithms attracted more attention from the machine learning community on the problem of algorithmic bias [82]. Some work studied biases emerging due to item popularity [117–120]. A recent work studied bias that is due to the assimilation bias in recommender systems [121]. Because recommender systems have a direct impact on humans, some recent research studied the impact of polarization on biasing rating data [122, 123] and proposed strategies to mitigate this polarization in collaborative filtering recommender systems [124], while other recent research pointed to bias emerging from continuous feedback loops between recommender systems and humans [125–128]. Overall, the study of algorithmic bias falls under the umbrella of fair machine learning [129].

Similar research has been conducted about how model updates in machine learning algorithms affect fairness. Sinha et al. aimed to understand how the feedback loop in recommender systems affects the entire rating matrix, and developed an algorithm to deconvolve the feedback loop with multiple assumptions [130]. Patro et al. proposed an ILP (Integer Linear Programming Problem)-based online optimization to deploy changes incrementally in multiple steps of recommendation so that the transition is smooth, and leads to an efficient and fair recommendation for both the producers and the customers in two-sided platforms [131]. Milano et al. focused on ethics problems within recommender systems updates, which results from how humans react to the output of recommender systems [132]. However, our paper focuses on the interaction between algorithms and humans, and how this interaction impacts humans’ ability to see(or discover) relevant items and how this in turn impacts the algorithms’ performance, rather than proposing a cure.

Taking all the above in consideration, we observe that most previous research has treated algorithmic bias as a static factor, which fails to capture the iterative nature of bias that is borne from continuous interaction between humans and algorithms. We argue that algorithmic bias evolves with human interaction in an iterative manner, which may have a long-term effect on algorithm performance and humans’ discovery and learning.

**Fig 3:**
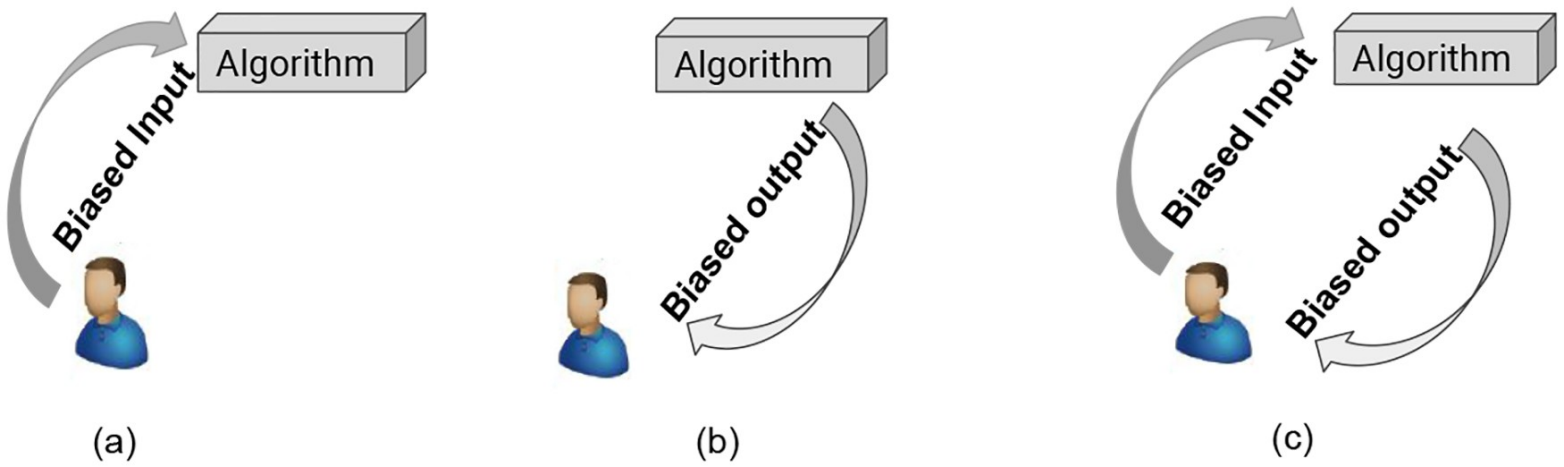
Evolution of bias between algorithm and human. In sub-figure (a), biased data from a human may lead to a biased algorithm, this is pre-algorithmic bias. In sub-figure (b), a biased algorithmic output might affect human behavior: For instance, by hiding certain items from humans, algorithms may affect human discovery, learning, and awareness in the long term. Sub-figure (c) indicates a continuous interaction between humans and algorithms that generates bias that we refer to as **iterated bias**, namely bias that **results from repeated interaction between humans and algorithms**.

In this study, we focus on simulating how the data that is selected to be presented to humans affects the algorithm’s performance and how human choice of action (specifically, to label or not to label the selected instance(s), that are presented to them by the algorithm), may in turn affect the algorithm’s performance (see Fig 3). In this work, we choose recommendation systems as the machine learning algorithm to be studied. One reason is that recommendation systems have more direct interaction options with humans, while information retrieval focuses on getting relevant information only. We further simplify the recommendation problem into a 2-class classification problem, e.g. like/relevant (class 1) or dislike/non-relevant (class 0), thus focusing on a personalized content-based filtering recommendation algorithm.

## Iterated algorithmic bias in online learning

Because we are interested in studying the interaction between machine learning algorithms and humans, we adopt an efficient way to observe the effect from both sides by using iterated interaction between algorithm and human action. Researchers from behavioral science have developed frameworks for investigating the effect of iterative interactions [69]. It is known that iterated interaction can be considered to generate Markov chains in the long-run, which gives us a well-formed framework to analyze the asymptotic effects of local decision [72, 74]. As stated before, we consider a simplified recommendation problem consisting of a 2-class classification problem. Thus we start with simple supervised machine learning to predict the ‘relevance’ class label of an item for a single user.

To begin, we consider three possible mechanisms for selecting information to present to users: **Random, Active-bias, and Filter-bias**. These three mechanisms simulate different regimes. Random selection is unbiased and used here purely as baseline for no filtering. Active-bias selection introduces a bias whose goal is to accurately predict user’s preferences. Filter-bias selection brings a bias whose goal is to provide relevant information or preferred items.

Before we go into the three forms of iterated algorithmic bias, we first investigate Pure Iterated Learning (PIL). We adopt some of the concepts from Griffiths [69]. Consider a task in which the algorithm learns a mapping from a set of *m* inputs *X* = {*x*_1_, …, *x*_*m*_} to *m* corresponding outputs {*y*_1_, …, *y*_*m*_} through a latent hypothesis *h*. For instance, based on previous purchase or rating data (*x*, *y*), a recommendation system will collect a new data about purchased item (*x*_*new*,_ *y*_*new*_) and update its model to recommend more interesting items to users. Here, *x* represents the algorithm’s selections and *y* represents people’s responses (e.g. likes/dislikes). Following Griffiths’ model for human learners, we assume a Bayesian model for prediction.

### Iterated learning with iterated filter-bias dependency

The extent of the departure that we propose from a conventional machine learning framework toward a human—machine learning framework, can be measured by the contrast between the evolution of iterated learning *without* and *with* the added dependency. As shown in Fig 2(a), without the dependency, the algorithm at step *n* accepts input point *x* from a set *X*, which is generated from a distribution *p*(*x*) that is independent of all other variables. Notation *q*(*x*) is used to represent this independence. Specifically, *q*(*x*) indicates an unbiased sample from the world, and not a selection made by the algorithms. On the other hand, with the dependency as shown in Fig 2(b), the algorithm, at iteration *n*, sees input *x*_*n*_ which is generated from both the objective distribution *q*(*x*) and another distribution *p*_*seen*_(*x*) that captures the dependency on the previous hypothesis *h*_*n*_. This in turns implies bias that con-affect of what can be seen by the user in the future. Thus, the probability of input item *x* is given by:

$$\begin{array}{c} p\left(x|h_{n}\right)=\left(1-\epsilon \right)p_{seen}\left(x|h_{n}\right)+\epsilon q\left(x\right) \end{array}$$ (1)

Recall that the probability of seeing an item is related to its rank in a rating based recommendation system or an optimal probabilistic information filter [2]. For a rating based recommendation system, the ranking is based on the prediction from the system, or the probability of relevance from prediction. In both situations, the selected data point *x* is likely to be highly rated or relevant, given *h*. In most circumstances, the recommendation system has a preferred goal, such as recommending relevant items (with y = 1). Then *x* will be chosen based on the probability of relevance *p*(*y* = 1|*x*, *h*_*n*_), *x* ∈ *X*. Assume that we have a candidate pool *X* at time *n* (In practice *X* would be the data points or items that the system can recommend at time *n*), then

$$\begin{array}{c} p_{seen}\left(x|h_{n}\right)=\frac{p(y=1|x,h_{n})}{\sum_{x\in X}p\left(y=1|x,h_{n}\right)} \end{array}$$ (2)

The selection of inputs depends on the hypothesis, and therefore information is not unbiased, *p*(**x**|*h*_*n*_)≠*q*(**x**). The derivations of the transition probabilities in Eq 2 will be modified to take into account Eq 1, and will become

$$\begin{array}{c} p\left(h_{n+1}|h_{n}\right)=\sum_{x\in X}\sum_{y\in Y}p\left(h_{n+1}|x,y\right)p\left(y|x,h_{n}\right)p_{seen}\left(x|h_{n}\right) \end{array}$$ (3)

Eq 3 can be used to derive the asymptotic behavior of the Markov chain with transition matrix *T*(*h*_*n*+1_) = *p*(*h*_*n*+1_|*h*_*n*_), i.e.

$$\begin{array}{c} p\left(h_{n+1}\right)=\epsilon p\left(h_{n+1}\right)+\left(1-\epsilon \right)T_{bias} \end{array}$$ (4)

$$\begin{array}{c} T_{bias}=[\sum_{x\in X}\sum_{y\in Y}p\left(h_{n+1}|x,y\right)\sum_{h_{n}\in H}p\left(y|x,h_{n}\right)p_{seen}\left(x|h_{n}\right)]p\left(h_{n}\right) \end{array}$$ (5)

Thus, iterated learning with filter bias converges to a mixture of the prior and the bias induced by filtering. To illustrate the effects of filter bias, we can analyze a simple and most extreme case where the filtering algorithm shows only the most relevant data in the next iteration (e.g. top-1 recommender). Hence

$$\begin{array}{c} x^{top}=arg\underset{x}{max}P\left(y|x,h\right) \end{array}$$ (6)

$$\begin{array}{c} p_{seen}\left(x|h_{n}\right)=\{\begin{array}{c} 1\;for\;x=x^{top}\\ 0\;\;\;otherwise\;\;\; \end{array}\} \end{array}$$ (7)

$$\begin{array}{c} T_{bias}=[\sum_{x\in X}\sum_{y\in Y}p\left(h_{n+1}|x,y\right)\sum_{h_{n}\in H}p\left(y|x_{n}^{top},h_{n}\right)]p\left(h_{n}\right). \end{array}$$ (8)

Based on Eq 3, the transition matrix is related to the probability of item *x* being seen by the user, which is the probability of belonging to class *y* = 1. The fact that $x_{n}^{top}$ maximizes *p*(*y*|*x*, *h*) suggests limitations to the ability to learn from such data. Specifically, the selection of relevant data allows the possibility of learning that an input that is predicted to be relevant is not, but does not allow the possibility of learning that an input that is predicted to be irrelevant is actually relevant. In this sense,**selection of evidence based on relevance is related to the confirmation bias in cognitive science**, where learners have been observed to (arguably maladaptively) select data which they believe to be true (i.e. they fail to attempt to falsify their hypotheses) [133]. **Put differently, recommendation algorithms may induce a blind spot where data that are potentially important for understanding relevance are never seen**.

### Iterated learning with iterated active-bias dependency

Active learning was first introduced to reduce the number of labeled samples needed for learning an accurate predictive model, and thus accelerate the speed of learning towards an expected goal [134, 135]. Instead of choosing random samples to be manually labeled for the training set, the algorithm can interactively query the user to obtain the desired data sample to be labeled [136].

$$\begin{array}{c} p_{active}\left(x|h\right)\propto 1-p\left(\hat{y}|x,h\right) \end{array}$$ (9)

where $\hat{y}=\arg\max_{y}(p(y|x,h))$. That is, **x** values are selected to be least certain about $\hat{y}$, the predicted *y* value.

Assuming a simplified algorithm where only the very uncertain data are selected, we can investigate the limiting behavior of an algorithm with the active learning bias. Assuming a mixture of random sampling and active learning, we obtain:

$$\begin{array}{c} x^{act}=\arg\max_{x}(1-p(\hat{y}|x,h)) \end{array}$$ (10)

$$\begin{array}{c} p\left(h_{n+1}\right)=\epsilon p\left(h_{n+1}\right)+\left(1-\epsilon \right)T_{active} \end{array}$$ (11)

Where

$$\begin{array}{c} T_{active}=[\sum_{x\in X}\sum_{y\in Y}p\left(h_{n+1}|x,y\right)\sum_{h_{n}\in H}p\left(y|x_{n}^{act},h_{n}\right)]p\left(h_{n}\right). \end{array}$$ (12)

The limiting behavior depends on the iterated active learning bias, $x_{n}^{act}$. This is, in most cases, in opposition to the goal of filtering, the algorithm will only select data point(s) which are closest to the learned model’s boundary, if we are learning a classifier for example. In contrast, the filtering algorithm is almost certain to pick items that it knows are relevant.

This is, of course, consistent with the different goals of recommendation and active learning. The analysis illustrates how the long-run implications of these different biases may be analyzed: By deriving the transition matrices implied by iterated application of data selection biases, we can see that both active learning and filtering have different goals, but focus on an ever more extreme (and therefore not representative) subset of data. Similar methods can be applied to more nuanced and interesting biases to shed light on the consequences of iterative interactions on the data.

### Iterated learning with random selection

The iterated random selection is considered as baseline for comparison purpose. This selection mechanism randomly choose instance to pass to next learner during iterations, i.e., the selection of the input *x* does not depend on the model learned.

$$\begin{array}{c} p\left(x|h_{n+1}\right)=p\left(x\right) \end{array}$$ (13)

### Iterated learning with human action bias

The above analysis assumes that people’s response is always observed. In the following, we extend our analysis to the more realistic case where users have a choice of whether to act or not on a given input.

Assume that people have some target hypothesis, h*, which represents optimal performance for the algorithm. Data are composed of an input provided by the algorithm, *x*, an output, *y*, and an action, *a*. The indicator variable *a* takes a value of 1 when people have provided a response, and a value of 0 when people have not. When the value of *y* is not observed, it is notated as *y* = *NULL*. These form triples ***d*** = (*x*, *y*, *a*)∈{(*x*_1_, *y*_1_, *a*_1_), …, (*x*_*n*_, *y*_*n*_, *a*_*n*_)}. The basic inference problem, from one iteration to the next, is then, *p*(*h*_*t*_|***d***) ∝ *p*(*d*|*h*_*t*−1_, h*)*p*(*h*_*t*−1_),

$$\begin{array}{c} p\left(h_{t}|d\right)\propto p\left(y|x,a,h^{*}\right)p\left(a|y^{*},x,h^{*}\right)p\left(x|h_{t-1}\right)p\left(h_{t-1}\right) \end{array}$$ (14)

where y* represents the output that would be observed, if an action were taken. The main change is in people’s choice of whether to respond, *p*(*a*|y*, *x*, h*). A missing at random assumption implies that *p*(*a*|y*, *x*, h*) does not depend on *x*, y*, or *h*, thus *p*(*a*|*x*, y*, h*) = *p*(*a*). If variables are missing due to a person’s choice, the probability of a missing value almost certainly depends on *x*, y* and/or h*. We can formalize this choice using Luce choice [56], a special case of softmax [137] (Note that both softmax and Luce choice have known issues for modeling human choice [56, 138]),

$$\begin{array}{c} p\left(a=1|y^{*},x,h^{*}\right)=\frac{U(a=1|y^{*},x,h^{*})}{U\left(a=0|y^{*},x,h^{*}\right)+U\left(a=1|y^{*},x,h^{*}\right)} \end{array}$$ (15)

where the choice of whether to act depends on the relative utility of acting as opposed to not acting. For example, if it is especially effortful to act, then people will be biased against acting. Alternatively, the utility of acting may depend on the value of y*. For example, it may be that there is greater perceived utility in acting when the value of y* is very low, as in the case of an angry customer or disappointed user. It is interesting to study the effect of biases in case of an imbalanced ability in acting as quantified by $\frac{p(a=1|y^{*}=1)}{p(a=1|y^{*}=0)}$, which we call imbalanced human action ratio.

In principle, one might think that this is related to the problem of dealing with missing data that is common in statistics [139]. Indeed, in our analyses, we showed one special case that reduces to the missing at random typically assumed in statistical applications [139]. However, the framework proposed here is in fact more general; it proposes a theory of why data are missing, and formalizes the problem as one of understanding human behavior [66, 140, 141].

### Evaluating the effect of iterated algorithmic bias on learning algorithms

In this section, we present the metrics we will use to measure the impact of different bias types on: (1) The discoverability of items: we define the blind spot metric for this purpose and this metric is novel; (2) The model: Thus we measure the boundary shift resulting from bias, which ends up being related to the proportion of items predicted as relevant by the model; (3) The inequality of the relevance predictions: We measure this using a standard metric for this purpose, namely the Gini coefficient.

#### Blind spot

The *blind spot* is defined as the set of data available to a relevance filter algorithm, for which the probability of being seen by the human interacting with the algorithm, that learned the hypothesis *h* is less than *δ*:

$$\begin{array}{c} D_{\delta }^{F}=\left\{x\in X|p_{seen}\left(x|h\right)<\delta \right\} \end{array}$$ (16)

In the real world, some data can be invisible to some users because of bias either from users or from the algorithm itself. Studying blind spots can enhance our understanding about the impact of algorithmic bias on humans. In addition, we define the *class-1-blind spot* or *relevant-item-blind spot* as the data in the blind spot, with true label *y* = 1

$$\begin{array}{c} D_{\delta }^{F+}=\left\{x\in D_{\delta }^{F}\;\text{and}\;y=1\right)\} \end{array}$$ (17)

Note that the blind spot in Eq 16 is also called *all-classes-blind spot*.

#### Boundary shifting

Boundary shifting indicates how different forms of iterated algorithmic bias affect the model *h* that is learned by an algorithm. It is defined as the number of points that are predicted to be in class *y* = 1 given a learned model *h*:

$$\begin{array}{c} b=\sum_{x\in X}^{}p\left(y=1|x,h\right) \end{array}$$ (18)

Here *b* is the number of points that are predicted as class *y* = 1 given a learned model *h*.

#### Gini coefficient

We also conduct a Gini coefficient analysis on how boundary shifts affect the inequality of predicted relevance for the test set. Let *p*_*i*_ = *p*(*y* = 1|*x*_*i*_, *h*). For a population with *n* values *p*_*i*_, *i* = 1 to *n*, that are indexed in non-decreasing order (*p*_(*i*)_ ≤ *p*_(*i*+1)_). The Gini coefficient can be calculated as follows [142]:

$$\begin{array}{c} G=(\frac{\sum_{i=1}^{n}\left(2i-n-1\right)p_{\left(i\right)}}{n\sum_{i=1}^{n}p_{\left(i\right)}}) \end{array}$$ (19)

The higher the Gini coefficient, the more unequal are the frequencies of the different labels. Given that the Gini coefficient measures the heterogeneity of the distribution of the relevance probabilities, it can be used to gauge the impact of different iterated algorithmic bias modes on the heterogeneity of the predicted probability in the relevant class during human-machine learning algorithm interaction.

## Research questions

The key issue is to show that information filtering may lead to systematic biases in the learned model, as captured by the classification boundary. Based on three metrics, we formulate three research questions:

**(RQ 1) How do different iterated algorithmic biases affect the behavior of models learned by a ML algorithm (with human action probability equal to 1)? We consider three aspects of a learned model to measure the outcome of algorithmic bias**:

**RQ 1.1) Boundary shift pre and post iteration (Eq 18)**;
**RQ 1.2) Gini coefficient of predicted testing set labels (Eq 19)**;
**RQ 1.3) Blind spot size pre and post iteration (Eq 17)**.

**(RQ 2) How does human action (whether to label data when requested to by the machine learning algorithm) affect the boundary shift? We consider the same three aspects to measure the effects as in RQ 1**.

**(RQ 3) Does human preference towards labeling relevant data affect the boundary? We consider the same three aspects to measure the effects as in RQ 1**.

## Experimental data sets

Our preliminary results are based on both synthetic and real data. As stated in Section Iterated Algorithmic Bias in Online Learning 1, we mainly focus on a two-class model of recommendation in order to perform our study. The classes are relevant/non-relevant, or like/dislike. In this situation, any classical supervised classification could be used in our model, such as Bayesian classifier [143], Logistic regression [144], Support Vector Machine (SVM) [145], or Extreme Learning Machine algorithms (ELM) [146, 147]. For the purpose of easier interpretation and visualization of the boundary and to more easily integrate with the probabilistic framework in Section Iterated Algorithmic Bias in Online Learning 1, we chose the Naive Bayes classifier.

**Fig 4:**
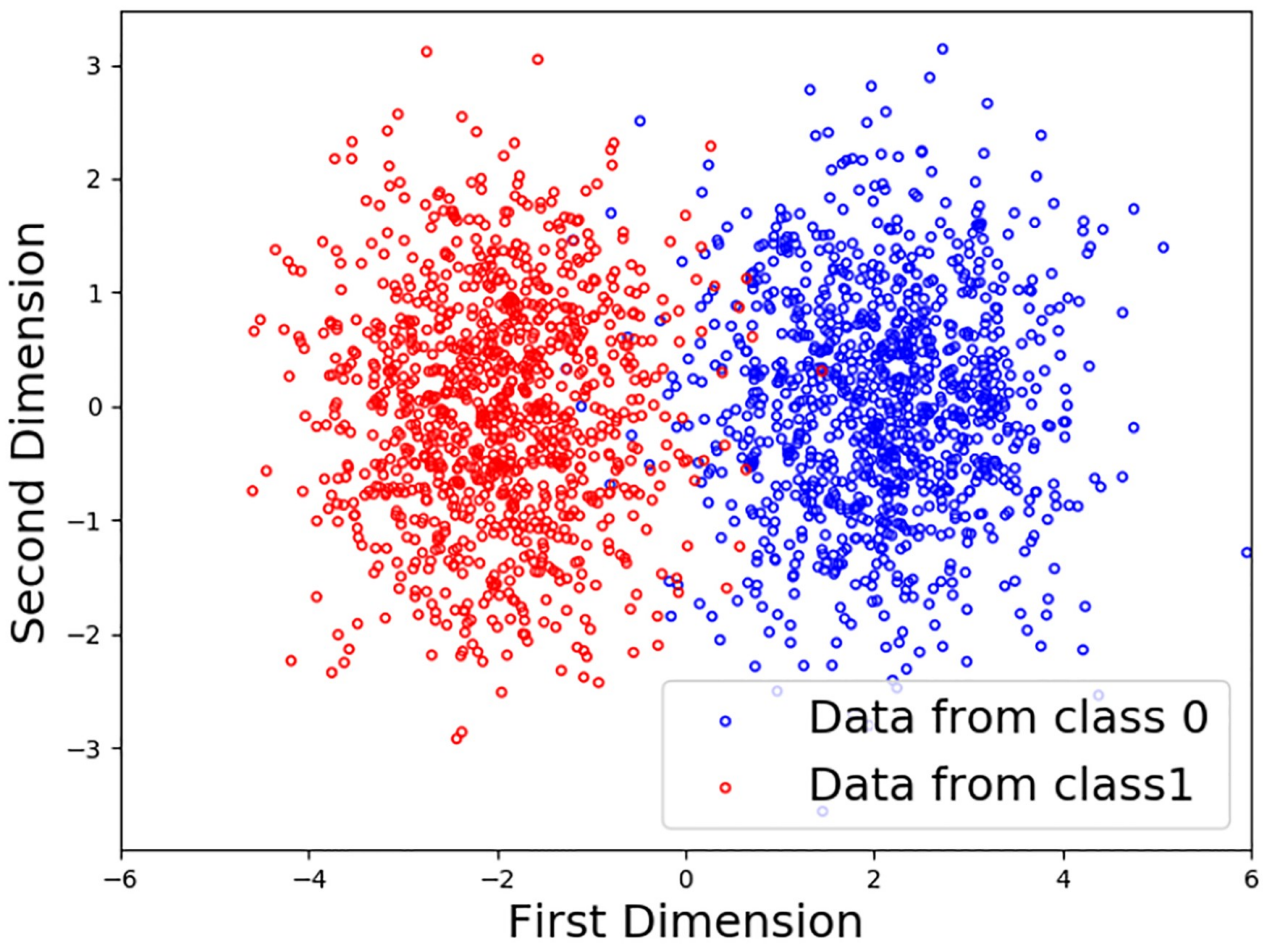
Original data with two classes.

**Synthetic data**: First, a 2D data set (see Fig 4) was generated from two Gaussian distributions corresponding to classes *y* ∈ {0, 1} for like (relevant) and dislike (non-relevant), respectively. Each class contains 1000 data points centered at {−2, 0} and {2, 0}, with standard deviation *σ* = 1. The data set is then split into the following parts:

- Testing set: used as a global testing set (200 points from each class).
- Validation set: used for the blind spot analysis (200 points from each class).
- Initializing set: used to initialize the first boundary (we tested different initializations with class 1/class 0 ratios as follows: 10/100;100/100;100/10).
- Candidate set: used as query set of data which will be gradually added to the training set (points besides from the above three groups will be added to the candidate set).

The reason why we need the four subsets is that we are simulating a real scenario with interaction between human and algorithm. Part of this interaction will include picking query data items and labeling them, thus augmenting the training set. Thus, to avoid depleting the testing set, we need to isolate these query items in the separate “candidate pool”. A similar reason motivates the remaining separate subsets in order to keep their size constant throughout all the interactions of module learning.

We are also motivated to run experiments on high dimensional synthetic data set. We thus generate 3D, 4D and 10D synthetic data to study the effects of different iterated algorithmic bias in Section 1.

**Real-life data set**: In addition to synthetic data, we are motivated to use a real data set that expresses likes/dislikes that could be used as a two-category data set similar to the synthetic data set. Here we use the movielens dataset [148], which contains 100,004 ratings on 9125 movies. These ratings were made by 671 users between January 09, 1995 and October 16, 2016. The latest dataset was generated on October 17, 2016. All 671 users had rated at least 20 movies. The ratings range in [1, 5] and include missing values. This data is a publicly available benchmark data of anonymous user ratings. No identifying demographic information is included. Each user is represented by an artificial id, and no other information is provided. The collection method complied with the terms and conditions of the website here http://files.grouplens.org/datasets/movielens/ml-latest-small-README.html. We discretized the ratings into two labels: the range [1, 3] was mapped to class 0, while (3,5] was mapped to class 1. The item content features were the movie genres. In order to perform similar experiments to the synthetic data simulations, we needed to focus on one user at a time. Thus we selected users who rated the highest number of movies and whose ratings are balanced between the two classes, analogously to our synthetic data. We first selected user *ID* = 547 to perform our study, then repeated the experiments on 7 more users. User 547 has rated 2312 movies throughout the 10 years. After removing some genres which appear across the movies less than 10 times, we end up with 18 valid genres. We then perform Principal Component Analysis (PCA) to reduce the dimensionality with component cutoff as 0.90 [149], and ended up with 11 content features. One reason we perform PCA is to be more consistent with the Naive Bayesian assumption that all features are independent.

**Methods**: The synthetic dataset represents a typical classification problem, it is the same for our real-life dataset after selecting one user and corresponding items. Each dataset contains two ground-truth categories of liked and disliked items. We wish to simulate the human-algorithm interaction at the heart of recommendation and information filtering. To do so, we consider three initialization possibilities: unbiased initialization in which examples are randomly selected from both categories in the same proportion; two types of biased sampling in which the relevant class (class 1) was oversampled by 10:1 or 1:10. Note that for the real-life data set, we only explore the unbiased initialization. We consider three forms of iterated algorithmic bias: random selection, active-bias which attempts to learn the true boundary between the two categories, and filter (or recommendation) bias which attempts to recommend only preferred items (see Section 1). We simulate different types of responses by the user as action probabilities that vary from labeling each item as it is recommended (human action probability of 1), to two cases where the user labels only some of the items provided by the algorithm (human action probabilities of 0.5 and 0.01). Note that absent any additional information, we assume action probabilities to be 1 for the real-life data set. We then simulate runs of 200 iterations where a single iteration consists of the algorithm providing a recommendation, the user labeling (or not) the recommendation, and the algorithm updating its model of the user’s preferences. Each combination of parameters yields a data set that simulates the outcome of the human and algorithm interacting. We simulate this whole process 40 times independently, which generates the data that we will use to investigate the research questions listed in Section Research Questions 1.

## Results

In this section, we present results using both synthetic and real-life data. We first present results from the 2D synthetic data described in Section Experimental Data Sets 1. We also explore high dimensional synthetic data in Section Experiments on High Dimensional Synthetic Data Set 1. The real-life data set experiments are presented in Section Experimental Data Sets 1. Finally, we summarize the conclusions from all our synthetic data experiments in Table 19, and from the real-life data experiments in Table 20.

### Experiments on 2D synthetic data

In this section, we presented results from the 2D synthetic data.

#### RQ 1: How does iterated algorithmic bias affect the learned categories?

To answer this question, we control for human action bias by assuming the data are labeled in each iteration, i.e. the probability of action is 1, *p*_*action*_ = 1. We adopt four different approaches to investigating this question. First, we will compare the inferred boundaries after interaction to the ground truth boundaries. Second, we will focus on the effects of iteration alone by analyzing the boundary before interaction and after. Third, We use the Gini coefficient to measure the heterogeneity or inequality of the predicted label distribution in the testing set. Fourth, we investigate the size of the blind spot induced by each of the iterated algorithmic bias modes. Together, these will describe the outcomes of algorithmic bias, in terms of how it interacts with initialization, and the consequences of algorithmic bias in terms of the induced blind spot.

#### RQ 1.1: Do different forms of iterated algorithmic bias have different effects on the boundary shift?

To answer this question, we control for human action bias by assuming the data are labeled in each iteration i.e. the probability of action is 1. And the initialization is balanced, i.e the ratio = 1:1. As shown in Eq 1, we here assume that *q*(*x*) is identical for all data points, thus we can ignore the second part of the equation, i.e. the probability of being seen is only dependent on the predicted probability of candidate points. Note that we could get some prior probability of *X*_*i*_, in which case we could add this parameter to our framework. Here, we assume them to be the same, hence we set *ϵ* = 0.

We wish to quantify differences in the boundary between the categories as a function of the different algorithm biases. To do so, we generate predictions for each test point in the test set by labeling each point based on the category that assigns it highest probability. We investigate the proportion of test points with the relevant label *y* = 1 at two time points: prior to human-algorithm interactions (immediately after initialization), and after human algorithm interactions.

We run experiments with each of the three forms of algorithm bias, and compare their effect on boundary shift. We also report the effect size based on *Cohen d* algorithm [150]. In this experiment, the effect size (ES) is calculated by *ES* = (*Boundary*_*t* = 0_ − *Boundary*_*t* = 200_)/*standard*.*dev*, here *standard*.*dev* is the standard deviation of the combined samples. We will use the same strategy to calculate the effect size in the rest of this paper. The results indicate significant differences for the filter bias condition (*p* <.001 by Mann-Whitney test or t-test, effect size = 1.96). In contrast, neither the Active Learning, nor the Random conditions resulted in statistically significant differences (*p* = .15 and.77 by Mann-Whitney test, or *p* = .84 and 1.0 by t-test; effective sizes.03 and 0.0, respectively).

**Fig 5:**
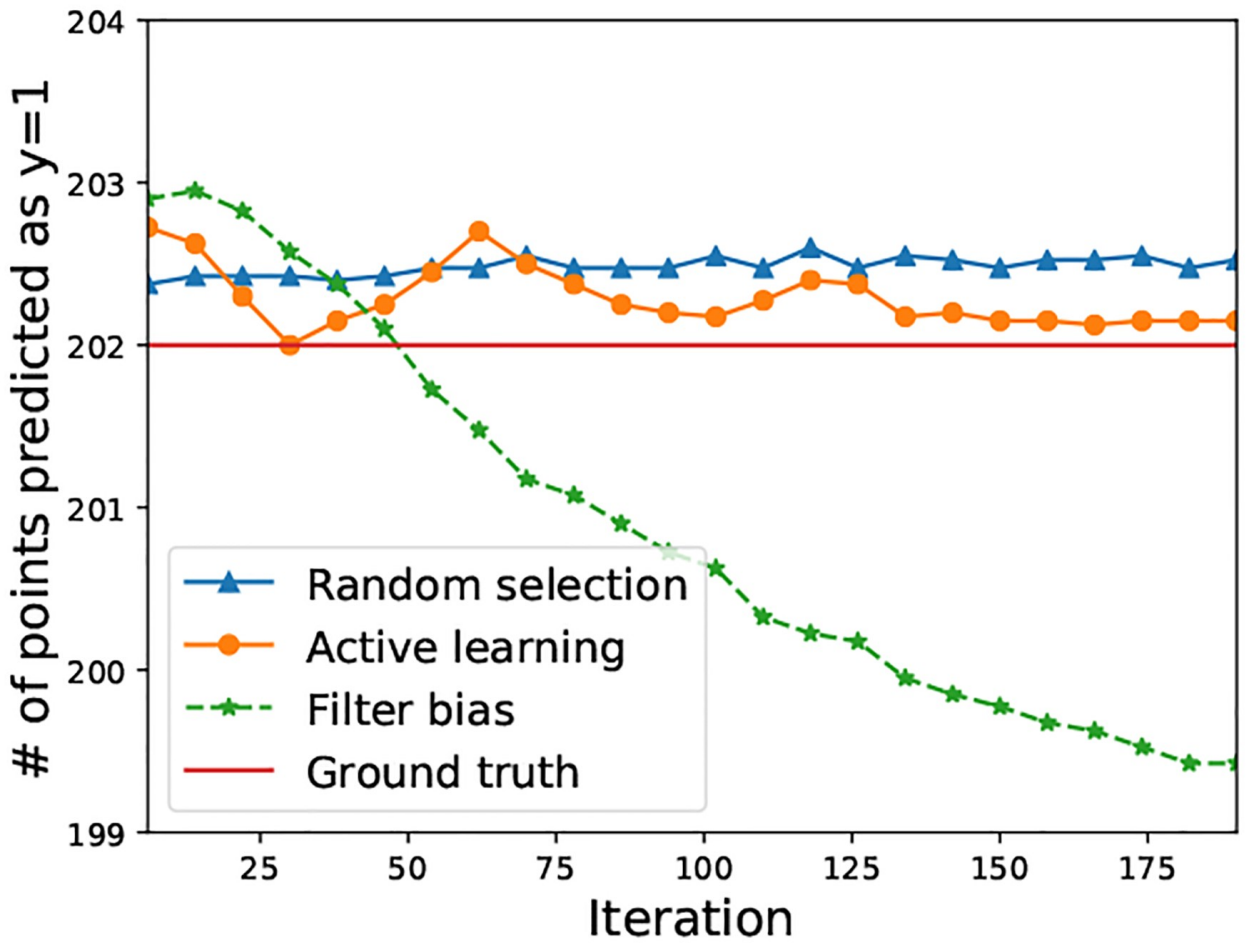
Boundary shift (Eq 18) based on the three iterated algorithmic bias forms. The y axis is the number of testing points which are predicted to be in class y = 1. This figure shows that random selection and active learning bias converge to the ground-truth boundary. Filter bias, on the other hand, results in decreasing numbers of points predicted in the target category class 1, consistent with an overly restrictive category boundary.

To illustrate this effect, we plot the number of points assigned to the target category versus ground-truth for each iterations. Fig 5 shows that random selection and active learning bias converge to the ground-truth boundary. Filter bias, on the other hand, results in decreasing numbers of points predicted in the target category class 1, consistent with an overly restrictive category boundary.

#### RQ 1.2: Do different iterated algorithmic bias modes lead to different trends in the inequality of predicted relevance throughout the iterative learning, given the same initialization?

In order to answer this question, we run experiments with different forms of iterated algorithmic bias, and record the Gini coefficient when a new model is learned and applied to the testing set during the iterations. We first run the Shapiro-Wilk normality test with all groups [151]. The p-value for filter bias, active learning bias and random selection are 0.91, 0.63 and 0.99 respectively. Therefore, we perform a one-way ANOVA tests [152].

**Fig 6:**
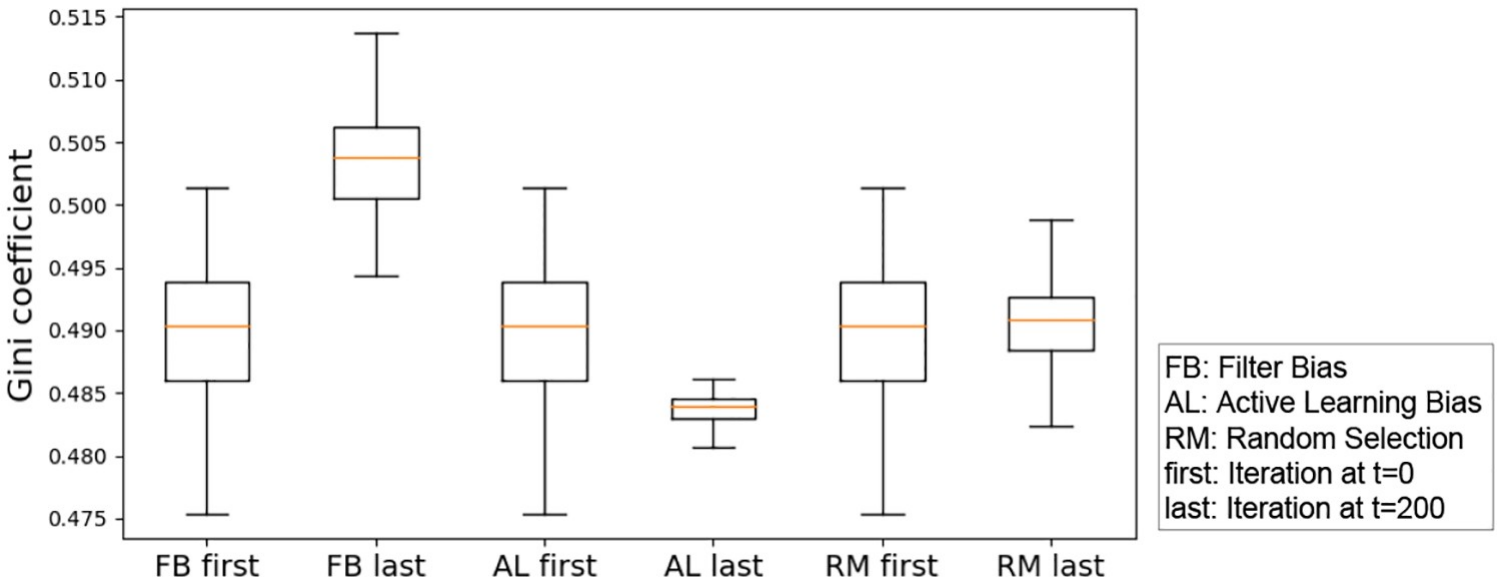
Box-plot of the Gini coefficient resulting from three forms of iterated algorithmic bias. An ANOVA test across these three iterated algorithmic bias forms shows that the Gini index values are significantly different. The p-value from the ANOVA test is close to 0.000 (<0.05), which indicates that the three iterated algorithmic bias forms have different effects on the Gini coefficient. Here, FB, AL and RM are the abbreviations of filter bias, active learning bias and random selection, respectively. The ‘first’ indicates the beginning of iteration (i.e., t = 0), while the ‘last’ means the end of iteration (i.e., t = 200). Note that we use these abbreviations in the rest of our paper.

Although the absolute difference between the first iteration and the last iteration is small (see Fig 6), a one-way ANOVA test across these three iterated algorithmic bias forms shows that the Gini index values are significantly different. The p-value from the ANOVA test is close to 1.0e-20 (<0.05), which indicates that the three iterated algorithmic bias forms have different effects on the Gini coefficient.

**Interpretation of this result**: Given that the Gini coefficient measures the inequality or heterogeneity of the distribution of the relevance probabilities, this simulated experiment shows the different impact of different iterated algorithmic bias forms on the heterogeneity of the predicted probability to be in the relevant class within human machine learning algorithm interaction. Despite the small effect, the iterated algorithmic bias forms affect this distribution in different ways, and iterated filter bias causes the largest heterogeneity level as can be seen in Fig 6.

#### RQ 1.3: Does iterated algorithmic bias affect the size of the class-1-blind spot and the all-classes-blind spot, i.e. is the initial size of the blind spot $D_{\delta }^{F}$ significantly different compared to its size in the final iteration?

The blind spot represents the set of items that are much less likely to be shown to the user. Therefore this research question studies the significant impact of an extreme filtering on the number of items that can be seen or discovered by the user, within human—algorithm interaction. If the size of the blind spot is higher, then iterated algorithmic bias results in hiding items from the user. In the case of the blind spot from class 1, this means that even relevant items are affected.

Recall from section 1 that some of the validation set data points have high probability to be seen, while others have low probability to be seen, the latter make up what we refer to as the blind spot. We study how iterated algorithmic bias affects the size of the blind spot. Here, *δ* is the threshold on the probability of being seen for an item to be considered in the blind spot. Recall that the blind spot items from class *y* = 1 are called *relevant item blind spot* or *class-1-blind spot* and the items from both classes are called *all-classes-blind spot*.

**Fig 7:**
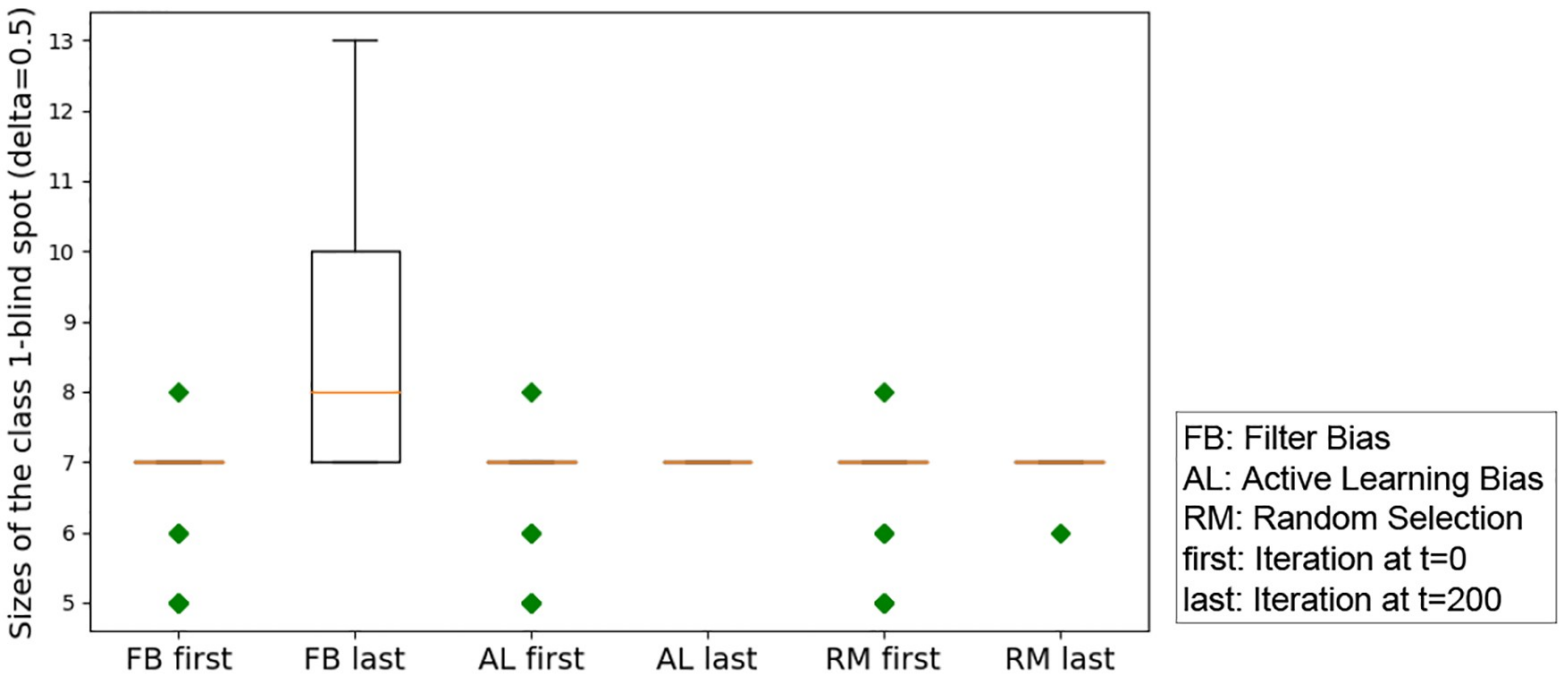
Box-plot of the size of the class-1-blind spot for all three iterated algorithmic bias forms. In this figure, the x-axis is the index of the three forms of iterated algorithms biases. As shown in this box-plot, the initial class-1-blind spot is centered at 7. This is because the 200 randomly selected initial points from both classes force the boundary to be similar regardless of the randomization.

We run experiments with *δ* = 0.5 for the class-1-blind spot, and record the size of the class-1-blind spots with three different iterated algorithmic bias forms. Here, we aim to check the effect of each iterated algorithmic bias form. Filter bias has significant effects on the class-1-blind spot, while random selection and active learning do not have a significant effect on the class-1-blind spot size (see Fig 7). The negative effect from iterated filter bias implies a large increase in the class 1 blind spot size, effectively hiding a significant number of `relevant' items. Table 2 summarizes the result of the statistical analysis. Note that the effect size is calculated by *ES* = (*BlindSpot*|_*t*=0_ − *BlindSpot*|_*t*=200_)/*standard:dev*.

**Table 2:** Results of the Mann-Whitney U test and t-test comparing the size of the class-1-blind spot for the three forms of iterated algorithmic bias. Bold means significance at p<0.05. The effect size is (*BlindSpot*|_*t* = 0_ − *BlindSpot*|_*t* = 200_)/*standard*.*dev*. The negative effect size shows that filter bias increases the class-1-blind spot size. For active learning bias, the p-value indicates the significance, however the effect size is small. Random selection has no significant effect.

	Filter Bias	Active Learning	Random Selection
Mann-Whitney test p-value	**2.4e-10**	**0.03**	0.06
t-test p-value	**2.2e-10**	**0.03**	0.06
effect size	**-1.22**	**-0.47**	-0.4

We perform a statistical test on the all-classes-blind spot with *δ* = 0.5. We first run the Shapiro-Wilk normality test. The p-values for the first and last iterations are respectively, 2.74e-7 and 0.037 for filter bias; 2.74e-7 and 1 for active learning bias; and 2.74e-7 and 2.9e-11 for random selection. Therefore, we perform a non-parametric statistical test on the pairs of data using the Mann-Whitney U test [153]. The p-value from the Mann-Whitney U test is close to 0.000 for filter bias. The negative effect from iterated filter bias implies a large increase in the all-class blind spot size, effectively hiding a significant number of ‘relevant’ and irrelevant items (see Table 3). For AL, the effect size has the opposite sign (positive) implying a significant decrease in hidden items (both relevant and non-relevant) based on the Mann-Whitney U test. Random selection results in no significant effect on the blind spot size.

**Table 3:** Results of the Mann-Whitney U test and t-test comparing the size of the all-classes-blind spot for the three forms of iterated algorithmic bias. The effect size is conducted as (*BlindSpot*|_*t* = 0_ − *BlindSpot*|_*t* = 200_)/*standard*.*dev*. The negative effect size shows that filter bias increases the class-1-blind spot size. On the other hand, both active learning and random selection have no significant effect.

	Filter Bias	Active Learning	Random Selection
Mann-Whitney test p-value	**5.9e-12**	0.5	0.47
t-test p-value	**1.4e-19**	0.18	0.13
effect size	**-1.44**	-0.3	-0.29

**Interpretation of this result**: Given that the blind spot represents the items that are much less likely to be shown to the user, this simulated experiment studies the significant impact of an extreme filtering on the number of items that can be seen or discovered by the user, within human-machine interaction. Iterated filter bias effectively hides a significant number of ‘relevant’ items that the user misses out on compared to AL. AL has no significant impact on the relevant blind spot, but increase the all-class blind spot to certain degree. Random selection has no such effect.

However, online systems which have a very wide set of options and where users tend to provide initial ratings for items that they like or see, do suffer from initial class imbalance. Class imbalance can also emerge from the algorithm intentionally asking users to rate only the most popular items, a common strategy used to collect initial ratings for new users. It is also important to notice that in different domains, initialization can have different imbalance patterns. For example in the popular Movielens-100k dataset [148], around 65% of users have rated more items with ratings higher than their own average rating. A similar phenomenon can be observed in other data sets such as Movielens-1M [148], Movelens-10M [148], Netflix Prize challenge dataset [154], and Book Crossings data set [155]. There are also users who have rated more items with lower ratings.

**Fig 8:**
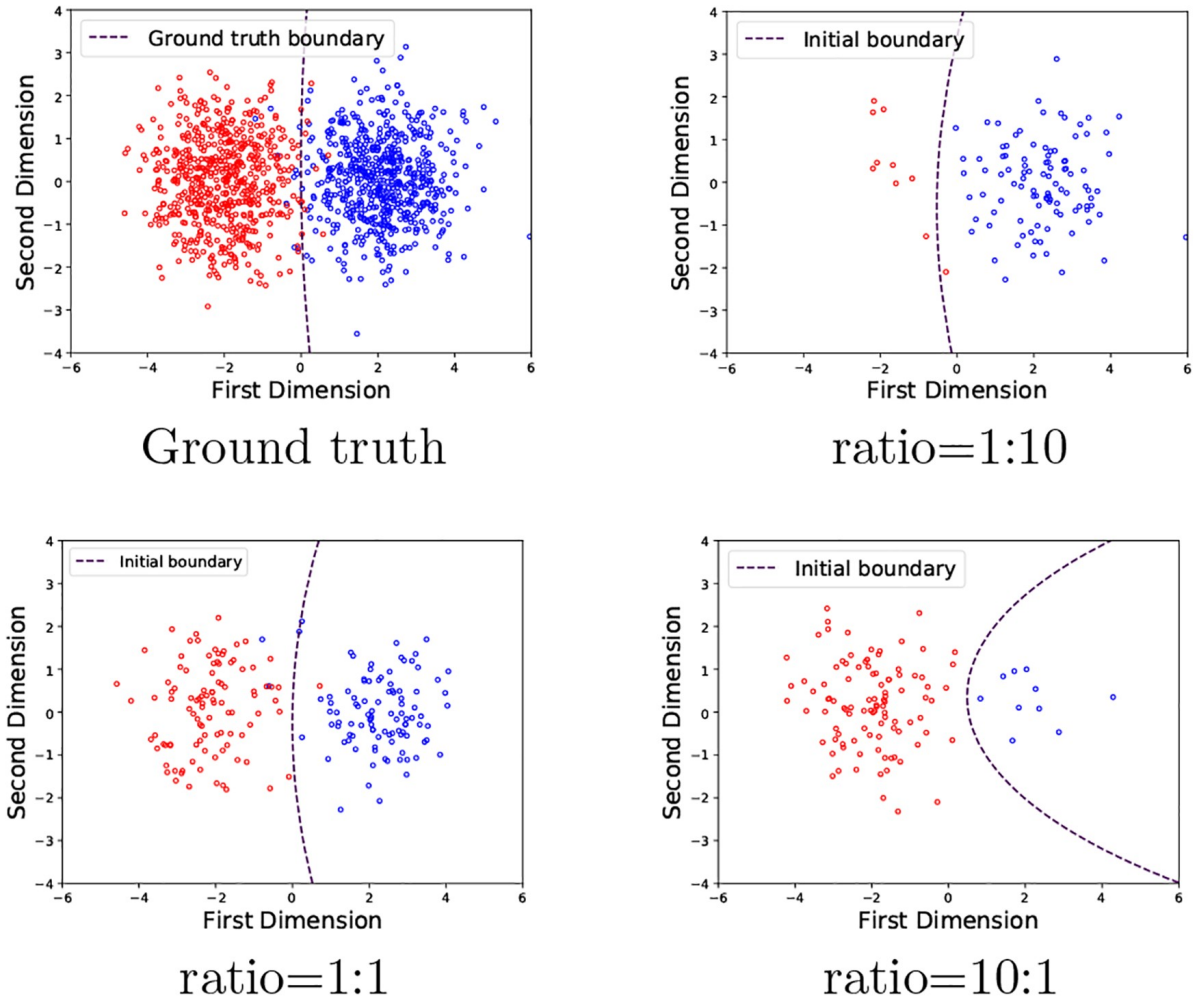
Different initialization imbalance ratios affect the starting model boundary ax expected.

Here, we measure how imbalanced initialization affects the algorithm’s performance. We set up three different class imbalance initialization ratios (class0: class1): 1:10, 1:1 and 10:1. Then we compare the learned boundaries with those three ratios. As shown in Fig 8, highly imbalanced class initialization leads to a bigger difference between the learned boundary and the ground-truth boundary. The ground-truth boundary is obtained from the Gaussian distributions that were used to generate the data points described in Section Experimental Data Sets 1. We also quantify the difference between the learned and true boundaries by measuring how initial boundaries predict the labels on the testing set. The accuracy of the ground-truth boundary and three imbalanced initial boundaries are recorded in Table 4. We can see that the increase in initialization imbalance ratio leads to lower accuracy on the testing set. Note that accuracies are averaged from 10 independent runs.

**Table 4:** Prediction accuracy of different imbalanced initializations ratio (class 1: Class 0) on the testing set and ground-truth boundary.

	ground truth	ratio = 1:1	ratio = 10:1	ratio = 1:10
accuracy	0.985	0.982	0.94	0.95

We wish to understand how imbalanced initialization affects the boundary shifting during the interactive learning process. To answer this question, we run experiments 40 times with the different class imbalanced initialization ratios and record the number of points which cross the boundary during iterative learning as well as the blind spot size. We will consider three imbalanced initialization (class 1: class 0) ratios, namely 10:1, 1:1 and 1:10.

**Fig 9:**
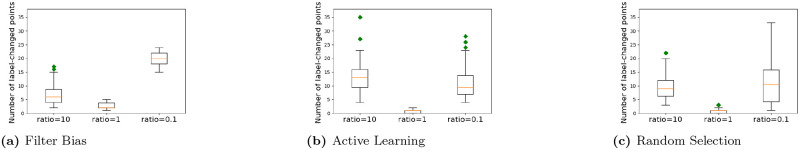
The box-plots showing the distribution of the number of points that moved across the boundary in the first and last iterations of the iterated learning for three iterated algorithmic biases with different class imbalanced initialization ratios. Because the number of points that move across the boundary indicates the intensity of the boundary shift, this shows that for all three iterated algorithmic bias modes, a high imbalanced initialization results in a higher boundary shift compared to a balanced initialization.

We record the number of points whose labels are different between the first iteration and last iterations, with imbalanced initialization ratio set to 10:1, 1:1 and 1:10. We first perform the Shapiro-Wilk normality test with all groups [151]. The p-value are 0.002, 0.001 and 0.06 for filter bias with four ratios; 0.01, 0.0003 and 0.001 for active learning bias; and 0.01, 0.0003 and 0.07 for random selection. Therefore, we perform a non-parametric statistical test using the Kruskal-wallis test [40] on each form of algorithmic bias. Fig 9 show the trends of label changes. The Number of label-changed points with ratio = 10:1 is higher than 2 for ratio = 1:1 in the iterated filter bias mode, and a similar result can be seen with iterated active learning bias and iterated random selection (see Fig 9). The p-values from the Kruskal-wallis test for all three forms of iterated algorithmic bias are 5.5e-19 (filter bias), 3.27e-17 (active learning bias) and 1.34e-15 (random selection), close to 0.00, which indicates that the class imbalance initialization affects the boundary shift for all three forms of bias. Also, the higher the class imbalanced initialization ratio, the more boundary shifting, as shown in Fig 9. On the other hand, the boundary shift of filter bias has a big difference when the ratio is 1:10, indicating that filter bias has a dramatic impact on the boundary shift when the imbalanced initialization ratio is high and more points are from the irrelevant class.

**Interpretation of this result**: Given that the number of label-changed points represents the number of items that move across the relevance boundary learned by the machine learning algorithm, this simulated experiment studies the impact of an initial class imbalance (a ratio of 10:1 or 1:10 versus a ratio of 1:1) when an extreme filtering strategy is used within human machine interaction.

In all cases and regardless of whether extreme filtering, AL, or random selection is used to collect feedback from the user, the initial class imbalance has a significant impact on the shift in the learned model’s boundary between relevant and non-relevant items. This test confirms that a more drastic initial class imbalance in the training data has a significant impact on the resulting boundary, and as a result on the judgment of items to be relevant or not by the learned model. Online systems which have a very wide set of options and where users tend to provide initial ratings for items that they like or see, do suffer from initial class imbalance. Class imbalance can also emerge from the algorithm intentionally asking users to rate only the most popular items, a common strategy used to collect initial ratings for new users.

#### RQ 2: Does human action bias affect the boundary?

In order to test how the human reaction affects the boundary shift, we set *p*_*action*_ = 1.0, 0.5 and 0.01, and record the results with different iterated algorithmic bias modes.

#### RQ 2.1: Does human action affect the boundary shift during iterative learning given a fixed iterated algorithmic bias mode?

We first want to compare the shift in the boundary induced by the different algorithmic bias forms, alone. We do so by analyzing the change in the boundary, i.e., the number of points in the test set which are predicted to be in class y = 1. We perform the Mann-Whitney U statistical test to see whether the different human action probabilities affect the boundary shift of the learned model for each iterated algorithmic bias and three possible human action probability levels. We also record results from the t-test. Effect size is also recorded between any pair of sets of boundary shift from the first iteration and last iteration. Table 5 shows that *p*_*action*_ affects the boundary shift more with iterated filter bias than with the other two forms of bias, which support the same conclusion as the previous experiment. The Mann-Whitney U test results agree with the t-test. Thus we conclude that human action affects the boundary shift: the more frequent human action is, the more significant is the effect on the boundary shift for only the iterated filter algorithmic bias. The other bias modes are not affected by different human action probabilities. Note that the effect size is calculated by the difference between the boundary of *t* = 0 and *t* = 200 divided by the standard deviation.

**Table 5:** Results of the Mann-Whitney U test and t-test for the boundary shift of the computed three forms of iterated algorithmic bias based on the different probability levels of human action. Here, effect size is *ES* = (*Boundary*|_*t* = 0_ − *Boundary*|_*t* = 200_)/*standard*.*dev*. Bold means the significance at p<0.05. The more the human reacts to the system, the larger is the boundary shift.

	Measurement	Filter Bias	Active Learning	Random
*p*_*action*_ = 1	Mann-Whitney test p-value	**0.4e- 11**	**0.9e- 5**	0.486
	t-test p-value	**7.1e- 14**	**0.002**	0.52
	effect size	**3.087**	**0.24**	-0.15
*p*_*action*_ = 0.5	Mann-Whitney test p-value	**1.8e- 6**	0.283	0.229
	t-test p-value	**1.0e- 5**	0.08	0.75
	effect size	**0.87**	0.19	0.08
*p*_*action*_ = 0.01	Mann-Whitney test p-value	0.259	0.336	0.42
	t-test p-value	0.38	0.6	0.9
	effect size	-0.27	0.14	-0.08

**Fig 10:**
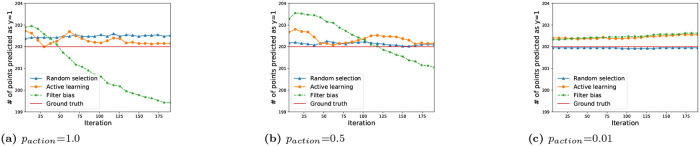
Effect of Human action on the boundary learned during the iterations. The y-axis is the number of testing points which are predicted to be relevant. We can see that for both random selection and active learning bias, the number of points predicted as relevant converges to the ground truth boundary when there is a high probability of human action. On the other hand, filter bias tends to diverge from the ground truth boundary in the presence of high human action probability. With *p*_*action*_ = 0.01, there are no obvious trends for all three algorithmic bias forms.

In addition, it is interesting to compare the final learned boundaries from the three iterated algorithmic bias forms with the ground truth. Therefore, we follow the same procedure as in RQ 1, comparing the learned boundary during the iterations with the ground truth boundary. Fig 10 shows the results for different iterated algorithmic bias modes with different human action probability. We can see that for both random selection and active learning bias, the number of points predicted as relevant converges to the ground truth boundary when there is a high probability of human action. On the other hand, filter bias tends to diverge from the ground truth boundary with high human action probability. With *p*_*action*_ = 0.01, there are no obvious trends for all three algorithmic bias forms.

#### RQ 2.2: Does human action affect the class-1-blind spot size during iterative learning given a fixed iterated algorithmic bias mode?

We want to compare the blind spot within each different iterated algorithmic bias mode, alone. We do so by analyzing the class-1-blind spot size. We run experiments with different human action probabilities and record the class-1-blind spot size comparing the blind spot sizes from the first iteration and last iteration. As shown in Table 6, with higher human action probability, the class-1-blind spot size is higher through all three iterated algorithmic bias modes.

**Table 6:** Results of the Mann-Whitney U test and t-test for the class-1-blind spot of the computed three forms of iterated algorithmic bias based on the different probability levels of human action. Here, effect size is *ES* = (*BlindSpot*|_*t* = 0_ − *BlindSpot*|_*t* = 200_)/*standard*.*dev*. Bold means the significance at p<0.05. The more the human reacts to the system, the bigger is the class-1-blind spot size.

	Measurement	Filter Bias	Active Learning	Random
*p*_*action*_ = 1	Mann-Whitney test p-value	**2.4e- 10**	**0.03**	0.06
	t-test p-value	**2.2e- 10**	**0.03**	0.06
	effect size	**-1.22**	**-0.47**	-0.4
*p*_*action*_ = 0.5	Mann-Whitney test p-value	**0.0002**	0.49	0.50
	t-test p-value	**7e- 5**	0.66	0.67
	effect size	**-0.8**	-0.1	-0.1
*p*_*action*_ = 0.01	Mann-Whitney test p-value	0.16	0.5	1.0
	t-test p-value	0.16	1	0.5
	effect size	0.02	0.0	0.0

#### RQ 2.3: Does human action affect the relevance prediction inequality or Gini coefficient during iterative learning given a fixed iterated algorithmic bias mode?

We wish to compare the inequality induced by each algorithmic bias form, alone. We do so by analyzing the Gini coefficient. We perform the Mann-Whitney U statistical test to see whether the human action will lead to different trends in the inequality of prediction. We run experiments with different human action probabilities, namely 1.0, 0.5 and 0.01, and compare the inequality between the first iteration and last iteration. Table 7 shows that higher human action probability leads to high inequality with the filter bias mode. On the other hand, active learning significantly decreases the inequality by querying points which are near the learned boundary.

**Table 7:** Results of the Mann-Whitney U test and t-test for the inequality of the computed three forms of iterated algorithmic bias based on the different probability levels of human action. Here, effect size is computed as *ES* = (*Gini*|_*t* = 0_ − *Gini*|_*t* = 200_)/*standard*.*dev*. Bold means the significance at p<0.05. The more the human reacts to the system, the bigger is the inequality in the prediction.

	Measurement	Filter Bias	Active Learning	Random
*p*_*action*_ = 1	Mann-Whitney test p-value	**2.5e- 14**	**3.8e- 9**	0.46
	t-test p-value	**5.2e- 35**	**2e-9**	0.68
	effect size	**-1.7**	**1.2**	-0.05
*p*_*action*_ = 0.5	Mann-Whitney test p-value	**3.5e- 26**	**1.84e-21**	0.46
	t-test p-value	**2.5e- 14**	**1.8e-14**	0.54
	effect size	**-1.3**	**1.78**	-0.07
*p*_*action*_ = 0.01	Mann-Whitney test p-value	0.16	0.14	0.45
	t-test p-value	0.17	0.001	0.22
	effect size	0.023	0.18	0.03

#### RQ 3: Does human preference towards labeling relevant data affect the boundary?

In research question 2 (RQ 2), we assumed that humans have a prior probability to act which is not dependent on the true label of the item presented. However, in a more realistic world, humans interact with the recommended items, according to their inner preference. In this section, we try to simulate this by assuming that humans have a higher probability to label or rate when the item presented is from relevant class y = 1 (see Eq 15). We therefore setup the class-dependent human action probability ratio to 10:1, i.e., *p*(*action*|*y* = 1) = 10*p*(*action*|*y* = 0).

#### RQ 3.1: Does human preference towards labeling relevant data affect the boundary shift during iterative learning given a fixed iterated algorithmic bias mode?

**Fig 11:**
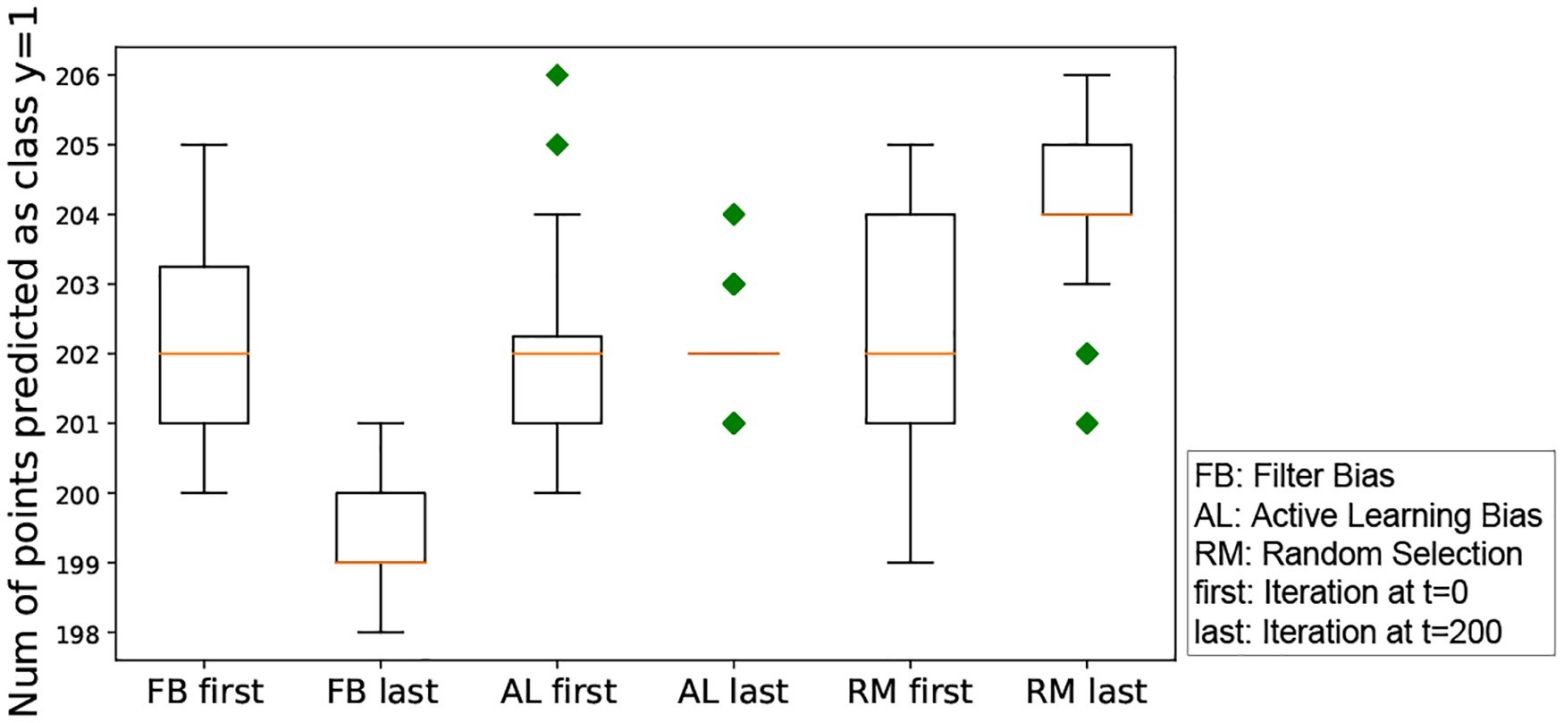
The box-plots showing the distribution of the number of points that are predicted to be in class y = 1 in the first and last iterations of the iterated learning for three iterated algorithmic biases with human action probability ratio 10:1. Filter bias significantly decreases the number of points predicted to be in class y = 1. On the other hand, random selection significantly increases the number of points predicted to be in class y = 1. Active learning has no such significant effect.

To answer this question, we run experiments and record the number of points which are predicted to be in class *y* = 1 before and after iterative learning. We first aim to understand how human preference changes the boundary. We do so by performing the Mann-Whitney test and t-test. Table 8 shows that the filter bias significantly decreases the number of points predicted to be in class y = 1. On the other hand, random selection significantly increases the number of points predicted to be in class y = 1. Active learning has no such significant effect. We also compare the effect across different iterated algorithmic bias modes by performing a Kruskal-wallis test. The p-value is 1.87e-21 (<0.01), which indicates that the bias modes have different effects on the boundary shift (see Fig 11).

**Table 8:** Results of the Mann-Whitney U test and the t-test comparing boundary shift for the three forms of iterated algorithmic bias with class-dependent human action probability ratio 10:1. The effect size is calculated as (*Boundary*|_*t* = 0_ − *Boundary*|_*t* = 200_)/*standard*.*dev*. The negative effect size for filter bias shows that it decreases the number of points which are predicted to be in class y = 1. Random selection increases the number of points, while active learning does not have a significant effect.

	Filter Bias	Active Learning	Random Selection
Mann-Whitney test p-value	**1.8e- 13**	0.05	**4.3*e* − 7**
t-test p-value	**6.4e- 24**	0.18	**6.1*e* − 13**
effect size	**1.6**	-0.2	**-1.1**

It is also interesting to compare the effect on boundary shift for different bias modes when the class-dependent human action probability ratios are set as 10:1 and 1:1. Table 9 shows the results. Both Filter bias and active learning biases show no significant difference in boundary shift between the two ratios. On the other hand, random selection has increased the number of points predicted to be in class y = 1 when the ratio was 10:1. The results are expected, since filter bias already prefers points from class y = 1. Active learning bias shows a smaller effect since this bias already prefers points that are close to the boundary. Random selection with class-dependent human action probability prefer points from class y = 1, however randomly. Therefore, it slightly shifts the boundary to class y = 0.

**Table 9:** Results of the Mann-Whitney U test and the t-test comparing boundary shift for the three forms of iterated algorithmic bias with class-dependent human action probability ratio 1:1 and 10:1. The effect size is calculated as (*Boundary*|_*ratio* = 1: 1_ − *Boundary*|_*ratio* = 10: 1_)/*standard*.*dev* at time t = 200. Both Filter bias and active learning bias show no significant difference between the two ratios. On the other hand, random selection leads to more points predicted to be in class y = 1.

	Filter Bias	Active Learning	Random Selection
Mann-Whitney test p-value	0.038	0.30	**7.8e- 9**
t-test p-value	0.07	0.18	**1.4e- 9**
effect size	-0.4	0.04	**-1.28**

#### RQ 3.2: Does human preference towards labeling relevant data affect the size of the blind spot during iterative learning given a fixed iterated algorithmic bias mode?

**Fig 12:**
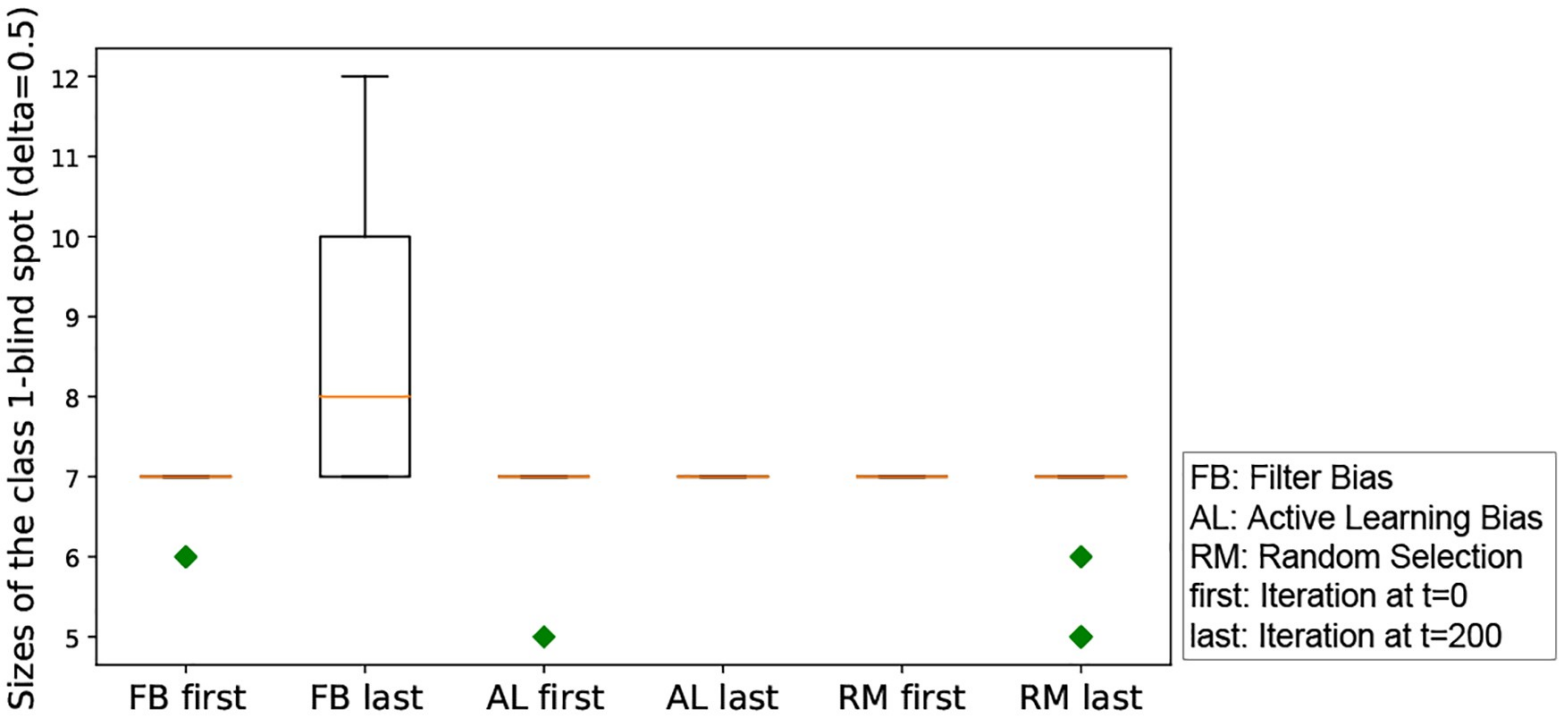
The box-plots showing the size of the class-1 blind spot in the first and last iterations of the iterated learning for three iterated algorithmic biases with human action probability ratio 10:1. Filter bias significantly decreases the number of points predicted to be in class y = 1. On the other hand, random selection significantly increases the number of points predicted to be in class y = 1. Active learning has no such significant effect.

To answer this question, we run experiments and record the size of the class-1 blind spot during iterative learning. Recall that the class-1 blind spot indicates how the interaction affects the human’s ability to discover items. We first aim to understand how human preference changes the blind spot size before and after iterative learning. We do so by performing the Mann-Whitney test and t-test. Table 10 shows that filter bias significantly increases the size of the class-1 blind spot. On the other hand, random selection significantly decreases the size of the class-1 blind spot. Active learning has no such significant effect. We also compare the effect across different iterated algorithmic bias modes by performing a Kruskal-wallis test. The p-value is 3.4e-15 (<0.01), which indicates that they have different effects on the boundary shift (see Fig 12).

**Table 10:** Results of the Mann-Whitney U test and the t-test comparing the size of class-1 blind spot for the three forms of iterated algorithmic bias with class-dependent human action probability ratio 10:1. The effect size is calculated as (*Boundary*|_*t* = 0_ − *Boundary*|_*t* = 200_)/*standard*.*dev*. The negative effect size for filter bias shows that it increases the size of the class-1-blind spot. Random selection decreases the blind spot size, while active learning does not have a significant effect.

	Filter Bias	Active Learning	Random Selection
Mann-Whitney test p-value	**1.1e- 10**	0.16	**0.0008**
t-test p-value	**1.2e- 9**	0.32	**0.002**
effect size	**-1.2**	-0.22	**0.7**

It is also interesting to compare the effect on the class-1 blind spot size with the class-dependent human action probability ratio set to 10:1 and 1:1. Table 11 shows the results for the class-1 blind spot size. Both Filter bias and active learning bias show no significant difference between the two ratios. On the other hand, random selection has increased the number of points predicted to be in class y = 1. Filter bias already prefers points from class y = 1 so they do not show any significant difference. Active learning bias has less effect since it prefers points that are close to the boundary. Random selection with class-dependent human action probability prefers points from class y = 1, however it is randomly. Therefore, it slightly shifts the boundary to class y = 0.

**Table 11:** Results of the Mann-Whitney U test and the t-test comparing boundary shift for the three forms of iterated algorithmic bias with class-dependent human action probability ratio 1:1 and 10:1. The effect size is calculated as (*Boundary*|_*ratio* = 1: 1_ − *Boundary*|_*ratio* = 10: 1_)/*standard*.*dev* at time t = 200. Both Filter bias and active learning bias show no significant difference between the two ratios. On the other hand, random selection decreases the class-1 blind spot size.

	Filter Bias	Active Learning	Random Selection
Mann-Whitney test p-value	0.7	1.0	**0.003**
t-test p-value	0.47	1.0	**0.004**
effect size	0.08	0.0	**0.64**

#### RQ 3.3: Does human preference towards to relevant class affect the inequality or Gini coefficient during iterative learning given a fixed iterated algorithmic bias mode?

**Fig 13:**
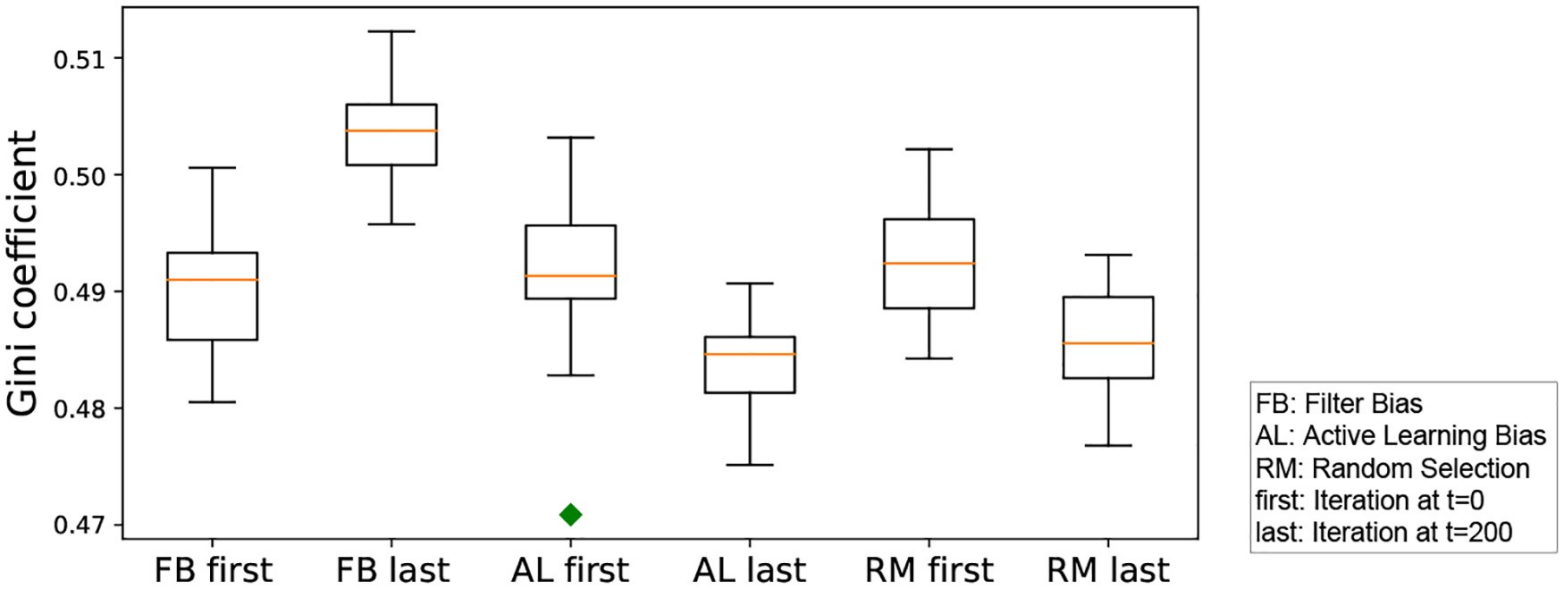
The box-plots showing the inequality score at the first and last iterations of the iterated learning for three iterated algorithmic biases with human action probability ratio 10:1. Filter bias significantly increases the Gini coefficient. On the other hand, random selection significantly decrease the Gini coefficient, as well as the active learning.

To answer this question, we run experiments and record the Gini coefficient before and after iterative learning. We first aim to understand how human preference affects the prediction inequality. We do so by performing the Mann-Whitney test and t-test. Table 12 shows that the filter bias significantly decreases the number of points predicted to be in class y = 1. On the other hand, random selection significantly increases the number of points predicted to be in class y = 1. Active learning has no such significant effect. We also compare the effect across different iterated algorithmic bias modes by performing a Kruskal-wallis test. The p-value is 3.3e-18 (<0.01), which indicates that they have different effect on the boundary shift (see Fig 13).

**Table 12:** Results of the Mann-Whitney U test and the t-test comparing the inequality of prediction for the three forms of iterated algorithmic bias with class-dependent human action probability ratio 10:1. The effect size is calculated as (*Gini*|_*t* = 0_ − *Gini*|_*t* = 200_)/*standard*.*dev*. The negative effect size for filter bias shows that it increases the inequality. Both active learning and random selection decrease the inequality.

	Filter Bias	Active Learning	Random Selection
Mann-Whitney test p-value	**8.2e- 14**	**4.6e- 14**	**1.3e-7**
t-test p-value	**3.3e- 34**	**1.7e- 13**	**2.9e-19**
effect size	**-1.6**	**1.31**	**1.84**

It is also interesting to compare the effect on the inequality of prediction with the class-dependent human action probability ratio set to 10:1 and 1:1. Table 13 shows the results for the inequality of prediction. Both Filter bias and active learning bias show no significant difference between the two ratios. On the other hand, random selection has increased the number of points predicted to be in class y = 1. There is no significant difference for the filter bias mode, since it already prefers points from class y = 1. Active learning bias has less effect since it prefers points that are close to the boundary. Random selection with class-dependent human action probability prefers points from class y = 1, however this occurs randomly. Therefore, it slightly decreases the inequality.

**Table 13:** Results of the Mann-Whitney U test and the t-test comparing the inequality for the three forms of iterated algorithmic bias with class-dependent human action probability ratio 1:1 and 10:1. The effect size is calculated as (*Boundary*|_*ratio* = 1: 1_ − *Boundary*|_*ratio* = 10: 1_)/*standard*.*dev* at time t = 200. Both Filter bias and active learning bias show no significant difference between the two ratios. On the other hand, random selection has decreased the class-1 blind spot size.

	Filter Bias	Active Learning	Random Selection
Mann-Whitney test p-value	0.47	0.11	**1.8e- 6**
t-test p-value	0.83	0.83	**1.6e- 8**
effect size	0.04	0.05	**1.06**

### Experiments on high dimensional synthetic data set

We perform similar experiments on 3D and 4D synthetic data using a similar data generation method. Our experiments produced similar results to the 2D data. We found that as long as the features are independent from each other, similar results are obtained to the 2D case above. One possible reason is that when features are independent, we can reduce them in a similar way to the 2D synthetic data set, i.e., one set of features that are highly related to the labels and another set of features that are non-related to the labels. Another possible reason is that independent features naturally fit the assumption of the Naive Bayes classifier. Finally, we generated a synthetic data with 10 dimensions, centered at (-2,0,0,0,0,0,0,0,0,0) and (2,0,0,0,0,0,0,0,0,0) with zero covariance between any two dimensions. We follow the same experimental procedure as the 2D synthetic data. Table 14 shows that the 10D synthetic data leads to similar results to the 2D synthetic data set. In order to avoid repetition, we here only report the results that show how different iterated algorithm bias modes affect the learned model during iterative learning. To conclude, repeated experiments on additional data with dimensionality ranging from 2 to 10 led to the same conclusions as the 2D data set.

**Table 14:** Experimental results with a 10D synthetic data set. The effect size is calculated by (*Measurement*|_*t* = 0_ − *Measurement*|_*t* = 200_)/*std*(⋅). The measurements are the three metrics presented in Section Evaluating the Effect of Iterated Algorithmic Bias on Learning Algorithms 1. We report the paired t-test results. For filter bias mode (FB), the results are identical to those of the 2D synthetic data across all three research questions. Active learning bias (AL) generates the same result as for the 2D synthetic data. Random selection (RM) has no obvious effect, similarly to the 2D synthetic data experiments.

	Bias type	Boundary Shift (p-value, ES)	Blind spot (p-value, ES)	Inequality (p-value, ES)
Statistical test	FB	**(8e-15, 1.4)**	**(3e-13, -1.4)**	**(1.8e-13, -1.6)**
	AL	(0.68, -0.09)	(0.5, 0.15)	**(1.8e-15, 1.63)**
	RM	(0.17, 0.17)	(0.1, -0.3)	(0.8, -0.01)

### Experiments on the real-life data set

We follow the same experimental procedure as for the synthetic data set from Section 1, but using the MovieLens data (as described in Section Experimental Data Sets 1). 200 items are randomly selected as testing set from both classes (like/dislike) for user 547. The testing set is also considered as validation set for the blind spot study. After that, 200 more items from the negative class (dislike) and 300 more items from the positive class (like) are randomly selected to initialize the first boundary. The reason we chose a different proportion from each is to be consistent with the proportions in the whole data set. The iterated learning commences after the initialization and continues for 200 iterations. With the real data, we aim to investigate how different iterated algorithmic biases affect the learned model. Thus, the human action probability is set to *p*_*action*_ = 1 and the preference to a certain class is not considered.

Visualizing the boundary in high-dimensional data is difficult. However most classifiers produce connected areas for each group [156]. We therefore can employ the number of data points which changed their label after applying the new model to intuitively capture the level of the boundary shift and to understand how the model is affected during the iterated learning process. By also studying the blind spot evolution, we can get a sense of the items that have a very low probability to be shown to the user, because they are part of the blind spot when considering the probability of belonging to class *y* = 1.

#### Boundary shift study

**Fig 14:**
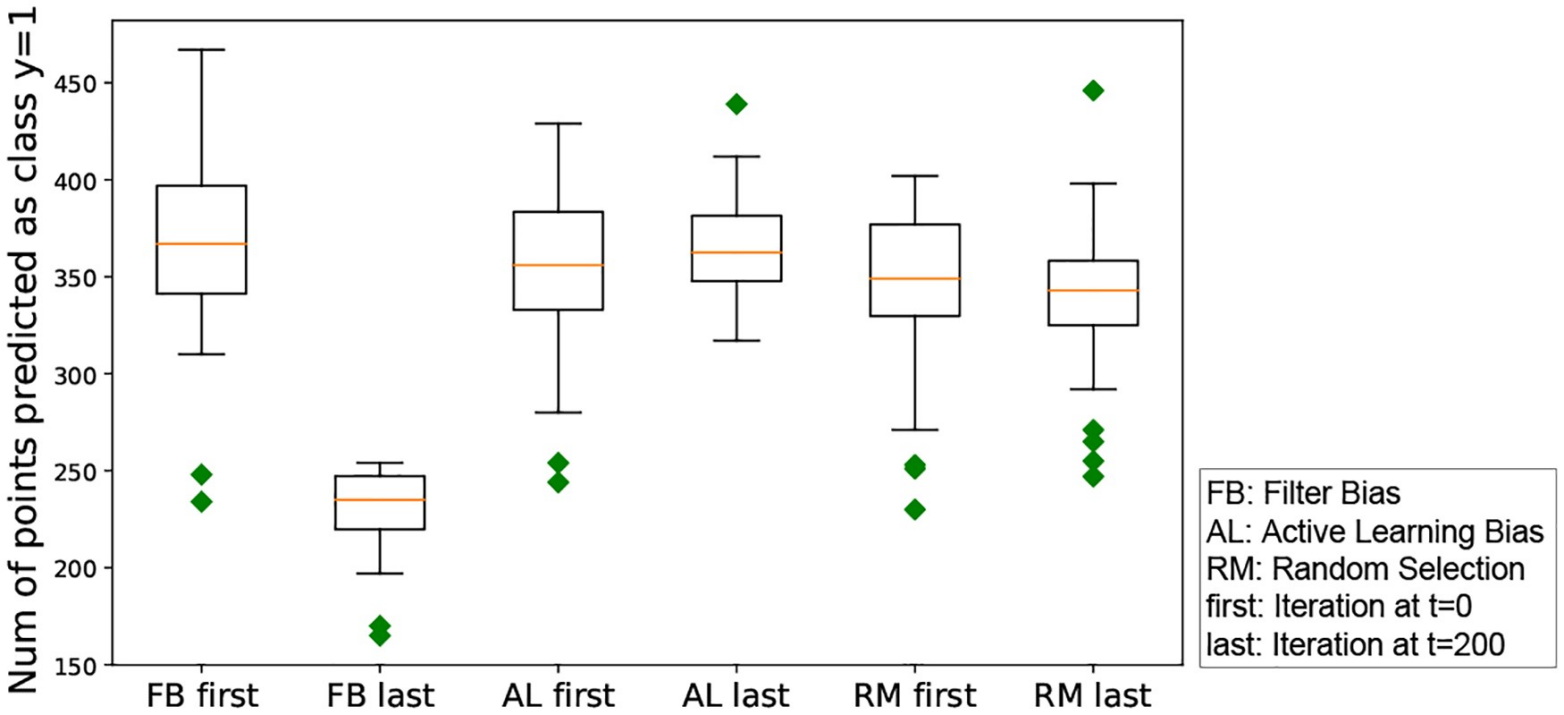
The box-plots showing the distribution of the number of points that are predicted to be in class y = 1 in the first and last iterations of the iterated learning for three iterated algorithmic biases with the MovieLens data set. Filter bias significantly decreases the number of points predicted to be in class y = 1. On the other hand, random selection and Active learning have no such significant effect.

Following the same procedure as in RQ 1 1, we run experiments for three different forms of iterated algorithmic bias and record the number of points which are predicted to be in class 1 in the first iteration and last iteration. We perform the Mann-Whitney U statistical test to see whether the different iterated algorithmic biases lead to different trends of the boundary shift, we also report the t-test results and effect size. Table 15 shows that there is a significant decrease in the number of points predicted to be in class y = 1 in the testing set for iterated filter bias. On the other hand, there is no significant difference with active learning bias and random selection. The effect size here is calculated using (*Boundary*|_*t* = 0_ − *Boundary*|_*t* = 200_)/*standard*.*dev*, we will use this setup for the rest of this section. It is also interesting that three different iterated algorithmic bias modes have different results of prediction given similar initialization (see Fig 14). All these results are consistent with the results from the synthetic data set.

**Table 15:** Results of the Mann-Whitney U test and the t-test comparing the boundary shift for the three forms of iterated algorithmic bias with the Movielens data set. The positive effect size indicates that filter bias leads to fewer points predicted to be in class y = 1. Random selection and active learning do not have a significant impact.

	Filter Bias	Active Learning	Random Selection
Mann-Whitney test p-value	**7.1e- 15**	**0.002**	0.42
t-test p-value	**7.6e- 25**	**0.0004**	0.84
effect size	**1.88**	**-0.57**	-0.04

We conclude that both iterated filter bias and iterated active learning bias have a significantly effect on the boundary shift, while random selection does not have a significant effect. This means that the nature of the model, and hence which items will be judged to be relevant to the user, changes depending on the iterated algorithmic bias, with filtering bias exerting the biggest influence. This kind of phenomenon was found to hold based on all the statistical tests performed in this paper for synthetic and real data.

#### Blind spot size study

**Fig 15:**
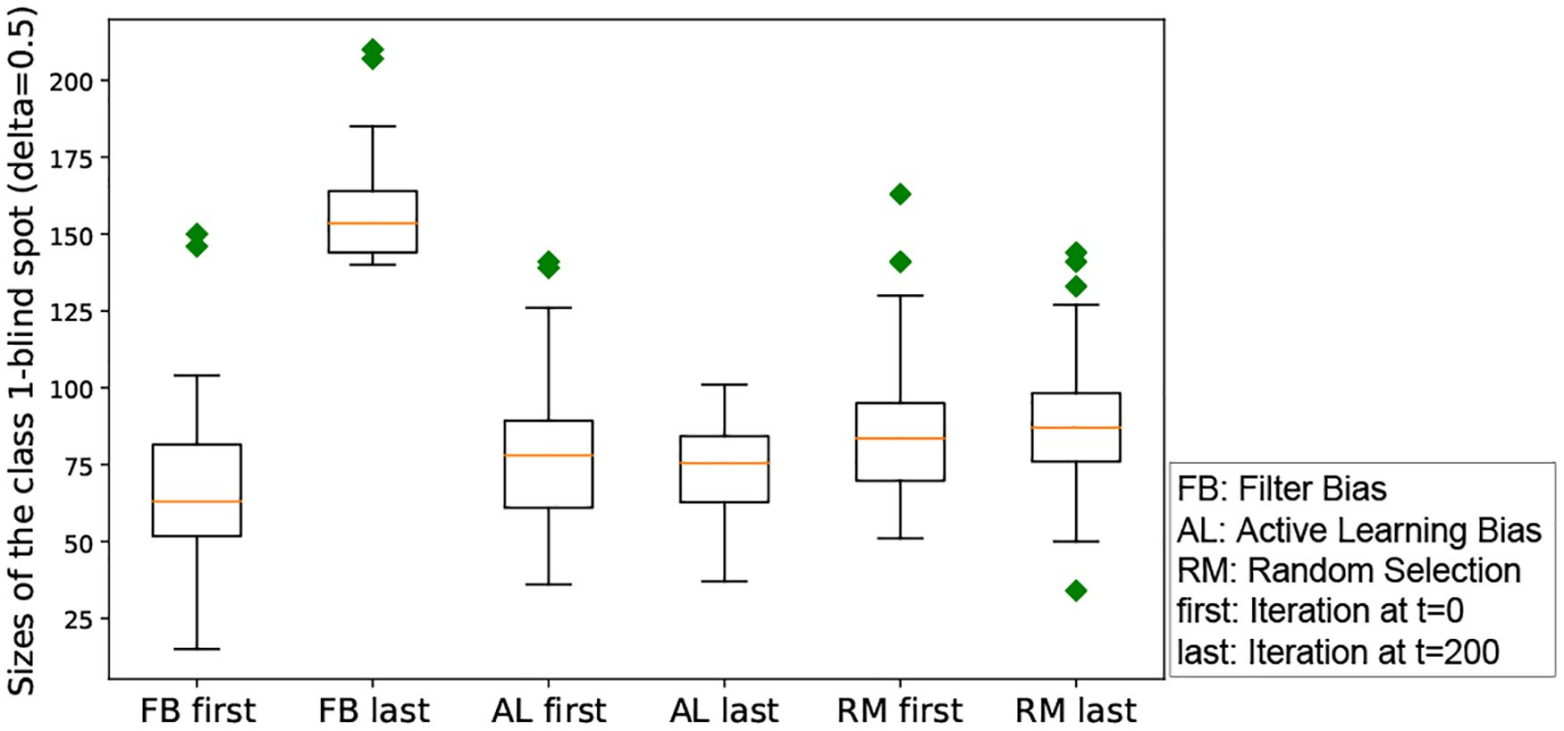
The box-plots showing the distribution of the size of the class-1 blind spot in the first and last iterations of the iterated learning for three iterated algorithmic biases with the MovieLens data set. Filter bias significantly increases the size of the class-1 blind spot. On the other hand, random selection and Active learning have no such significant effect.

In order to test how different iterated algorithmic bias modes affect the blind spot size, we ran experiments for three different forms of iterated algorithmic bias and recorded the size of the blind spot in the first iteration and last iteration. We performed the Mann-Whitney U statistical test and we also report the t-test results and effect size. Table 16 shows that there is a significant increase in the class-1 blind spot sizes in the testing set for iterated filter bias and active learning bias. On the other hand, there is no significant difference with random selection (see Fig 15).

**Table 16:** Results of the Mann-Whitney U test and the t-test comparing the size of the class-1-blind spot for the three forms of iterated algorithmic bias with the Movielens data set. The negative effect size indicates that filter bias leads to a bigger blind spot. Both random selection and active learning do not have a significant impact.

	Filter Bias	Active Learning	Random Selection
Mann-Whitney test p-value	**7.0e- 15**	**0.0002**	0.15
t-test p-value	**4.2e- 26**	**0.0001**	0.46
effect size	**-1.89**	**0.66**	0.16

**It is important to note that this impact is significant enough to result in hiding even relevant items from the user**. A similar impact was found when the initial labeled (training) set is class imbalanced with more relevant items than non relevant items. **Optional human willingness to label items seems to have a significant impact on the resulting model and thus on which items are judged to be relevant and in turn can be discovered by the user**.

#### Inequality study

**Fig 16:**
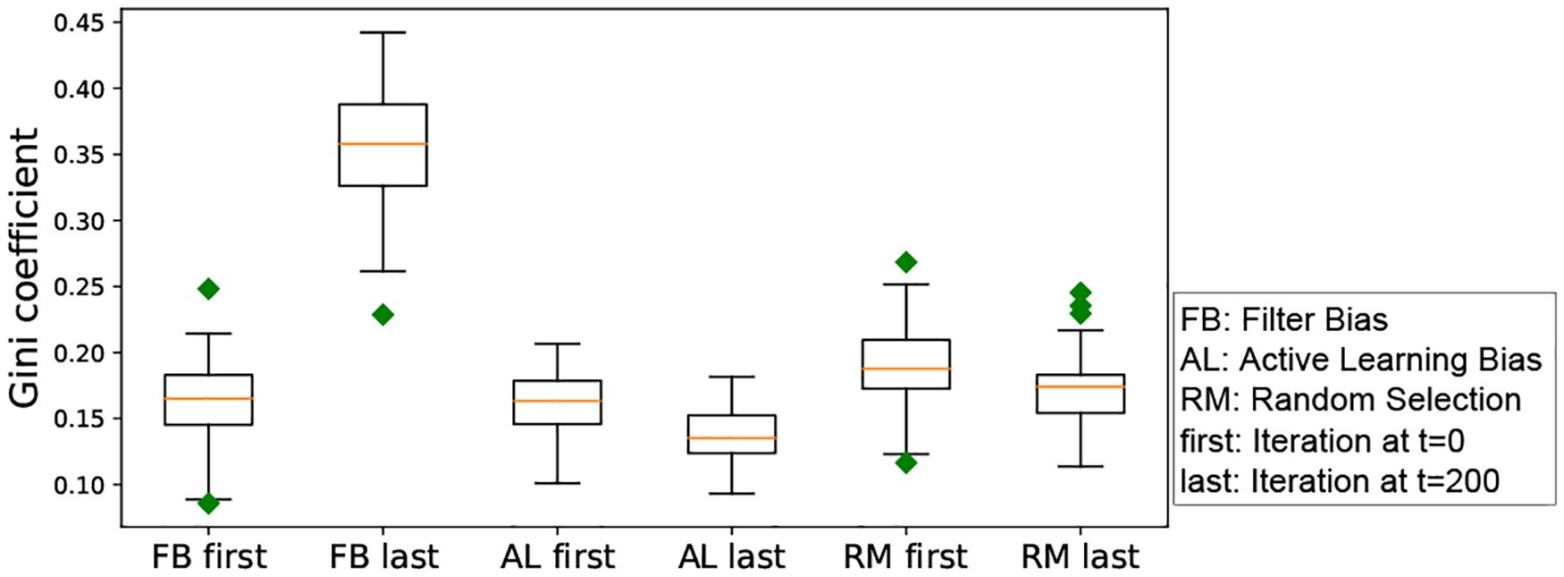
The box-plots showing the distribution of the Gini coefficient of the prediction in the first and last iterations of the iterated learning for three iterated algorithmic biases on the MovieLens data set. Filter bias significantly increases the in equality of prediction. On the other hand, random selection and Active learning lead to a significantly decrease in the inequality of prediction.

In order to test how different iterated algorithmic bias modes affect the inequality of predictions, we ran experiments for three different forms of iterated algorithmic bias and recorded the Gini coefficient in the first iteration and last iteration. We performed the Mann-Whitney U statistical test and we also report the t-test results and effect size. Table 17 shows that filter bias leads to a significant increase in the Gini coefficient, while both active learning and random selection result in a significant decrease in the Gini coefficient. It is important to note that both random selection and active learning are used to build an accurate model in machine learning. Therefore, both bias modes decrease the inequality (see Fig 16).

**Table 17:** Results of the Mann-Whitney U test and the t-test comparing the inequality of prediction for the three forms of iterated algorithmic bias with the Movielens data set. The negative effect size indicates that filter bias leads to high inequality of relevance prediction. Both random selection and active learning significant decrease on the inequality.

	Filter Bias	Active Learning	Random Selection
Mann-Whitney test p-value	**7.7e- 15**	**1.9e-5**	**0.008**
t-test p-value	**7.4e- 25**	**1.7e-9**	**4.4e- 5**
effect size	**-1.83**	**0.95**	**0.5**

We also ran experiments with other 7 users. They all produced similar results (see Table 18).

**Table 18:** Top 8 most active users in MovieLens data set and statistical analysis results with paired t-test. The effect size is calculated as (*Measurement*|_*t* = 0_ − *Measurement*|_*t* = 200_)/*standard*.*dev*. Bold means significance, and ES means the effect size. Filter bias has a consistently significant and sizable effect on the three measurements across all 8 users. Random selection has less impact on the boundary shift and blind spot. However it significantly decreases the inequality. Active learning aims to help learn correct boundary, therefore it highly depends on the initial data points. Active learning affects points close to the boundary, thus it has limited effects overall.

	Total movies rated	Positive rated	Negative rated	Bias type	Boundary Shift (p-value, ES)	Blind spot (p-value, ES)	Inequality (p-value, ES)
User ID = 547	2391	1409	982	FB	**(7.6e-25, 1.88)**	**(4.2e-26, -1.89)**	**(7.4e-25, -1.83)**
				AL	**(0.0004, -0.57)**	**(0.001, 0.66)**	**(1.7e-9, 0.95)**
				RM	(0.84, -0.04)	(0.46, 0.16)	**(4.4e-5, 0.5)**
User ID = 564	1868	1115	753	FB	**(1.0e-14, 1.37)**	**(1.1e-12, -1.45)**	**(1.5e-21, -1.67)**
				AL	**(1.7e-9, -0.99)**	**(3e-9, 0.95)**	**(9e-12, 0.92)**
				RM	**(0.004, -0.48)**	**(0.008, 0.45)**	**(2.6e-6, 0.73)**
User ID = 624	1735	1043	692	FB	**(2.3e-7, 0.8)**	**(2.7e-7, -1.28)**	**(8.2e-26, -1.7)**
				AL	**(8.4e-11, -1.28)**	**(7.8e-11, 1.29)**	**(7.5e-17, 1)**
				RM	**(2.8e-5, 0.64)**	**(1.6-5, 0.7)**	**(8e-6, 0.64)**
User ID = 15	1700	857	843	FB	**(3.4e-18, 1.4)**	**(2.3e-18, -1.5)**	**(2.6e-30, -1.76)**
				AL	**(4.3e-8, 0.93)**	**(7.4e-9, 0.9)**	**(0.0003, 0.35)**
				RM	**(1.6e-6, 0.73)**	**(2.1e-6, -0.7)**	(0.16, -0.17)
User ID = 73	1610	1016	594	FB	**(1.8e-8, 1.02)**	**(1.6e-8, -1.0)**	**(7.6e-36, -1.81)**
				AL	**(1.7e-12, -1.5)**	**(5.4e-11, 1.46)**	**(9.3e-24, 1.42)**
				RM	**(2e-7, -0.9)**	**(5.6e-7, 0.85)**	**(6.4e-7, 0.66)**
User ID = 452	1340	613	727	FB	**(0.001, 0.46)**	**(0.0006, -0.7)**	**(1.1e-12, -1.31)**
				AL	**(2.8e-8, 1.2)**	**(5.9e-10, -1.24)**	(0.78, 0.34)
				RM	**(0.0101, 0.5)**	**(0.009, -0.52)**	(0.7, 0.04)
User ID = 468	1291	795	496	FB	**(5.2e-10, 1.18)**	**(8.8e-12, -1.29)**	**(3.3e-26, -1.74)**
				AL	**(1.8e-18, -1.6)**	**(3.1e-18, 1.61)**	**(1.9e-26, 1.67)**
				RM	**(5.6e-12, -1.36)**	**(3.2e-13, 1.44)**	**(1.1e-13, 1.15)**
User ID = 380	1063	620	443	FB	**(3.2e-11, 1.22)**	**(2.6e-11, -1.26)**	**(2.3e-27, -1.76)**
				AL	**(1.36e-13, -1.32)**	**(1.13e-12, 1.28)**	**(4.7e-15, 0.96)**
				RM	**(1.3e-9, -0.96)**	**(1.53e-5, 0.89)**	**(0.002, 0.7)**

## Conclusion

We investigated three forms of iterated algorithmic bias (filter, active learning, and random baseline) and how they affect the performance of machine learning algorithms by formulating research questions about the impact of each type of bias. Based on statistical analysis of the results of several controlled experiments using synthetic and real data, we found that (see the overall synthesis of findings in Tables 19 and 20):

1. The three different forms of iterated algorithmic bias (filter, active learning, and random selection, used as query mechanisms to show data and request new feedback/labels from the user), **do affect algorithm performance** when fixing the human interaction probability to 1. Different initial class imbalance in the training data used to generate the initial relevance boundary, significantly affect the machine learning algorithm’s results for all three forms of iterated algorithmic bias, **impacting boundary shift which hides relevant items (class 1-blind spot)**.
2. Iterated filter bias has a more significant effect on the class-1-blind spot size compared to the other two forms of algorithmic biases. **This means that iterated filter bias, which is prominent in personalized user interfaces, can limit humans’ ability to discover data that is relevant to them**.
3. The iterated learning framework is effective for analyzing the impact of iterated algorithmic bias in human-algorithm interaction.

**Table 19:** Summary of research question (RQs) and findings for synthetic data.

RQ	Sub Questions	Main Findings
1	RQ1.1: Do different forms of iterated algorithmic bias have different effects on the boundary shift? RQ1.2: Do different iterated algorithmic bias modes lead to different trends in the inequality of predicted relevance throughout the iterative learning? RQ1.3: Does the iterated algorithmic bias affect the size of the class-1-blind spot?	Filter bias reduces the # of items predicted as class y = 1 and increases the blind spot size, which indicates it will limit the users’ ability to discover new items. Filter bias increases the inequality of prediction, which leads to even more inequality. Random selection and active learning bias show no/little effect on the blind spot. Highly imbalanced class initialization leads to a bigger difference between the learned boundary and the ground-truth boundary.
2	RQ2.1: Does human action affect the boundary shift during iterative learning? RQ2.2: Does human action affect the class-1-blind spot size during iterative learning given a fixed iterated algorithmic bias mode? RQ2.3: Does human action affect the relevance prediction inequality (or Gini coefficient)?	Human action affects the boundary shift more with iterated filter bias than with the other two forms of bias. For both random selection and active learning bias, the number of points predicted as relevant converges to the ground truth boundary when there is a high probability of human action. Filter bias tends to diverge from the ground truth boundary with high human action probability. The more humans react to the recommender system, the higher the impact of each iterated algorithmic bias mode.
3	RQ3.1: Does human preference towards labeling relevant data affect the boundary shift during iterative learning? RQ3.2: Does human preference towards labeling relevant data affect the size of the blind spot during iterative learning? RQ3.3: Does human preference towards to relevant class affect the inequality or Gini coefficient during iterative learning?	For both filter bias and active learning, the number of points predicted as class y = 1, with and without the class-dependent human action probability, show no significant difference. For both filter bias and active learning, the size of the class-1 blind spot, with and without the class-dependent human action probability, have no significant difference. Random selection decreases the class-1 blind spot size, and increases the number of points predicted as class y = 1, with the class-dependent human action probability.

**Table 20:** Summary of research question (RQs) and findings for real data.

Research Question	Main Findings
• How do different iterated algorithmic bias modes affect the boundary shift?	• Both iterated filter bias and iterated active learning bias have a significantly effect on the boundary shift, while random selection does not have a significant effect, indicating that the nature of the model, and hence which items will be judged to be relevant to the user, changes depending on the iterated algorithmic bias, with filtering bias exerting the biggest influence.
• How do different iterated algorithmic bias modes affect the blind spot size?	• There is a significant increase in the class-1 blind spot size in the testing set for iterated filter bias and active learning bias. On the other hand, there is no significant difference with random selection.
• How do different iterated algorithmic bias modes affect inequality of prediction?	• Filter bias leads to a significant increase in the Gini coefficient, while both active learning and random selection show a significant decrease in the Gini coefficient.

There is a long tradition in machine learning algorithms whose performance is guaranteed in the context of unbiased data. Similarly, there is a long tradition in the psychology of human learning of treating learning as inference from unbiased data. Humans and AI algorithms are both increasingly intertwined in feedback loops that end up feeding biased data to both humans and algorithms. Our research attempted to better understand how algorithm performance and human behavior depend on one another and how those dependencies affect long run performance. Our ongoing work will pave the road for a framework in which the study of human-algorithm interaction may progress.

### Future work

Future work will consider the following three directions: 1) extending our framework and experiments to more users in a collaborative filtering setting; 2) taking into account additional parameters and configurations when testing the impact of iterated algorithmic bias and human interaction on ML models, including: (A) changing the number of items recommended in top N relevant item recommendation lists and (B) applying different types of AL (we only investigated uncertainty-based AL); 3) human experiments that study research questions that are similar to the ones formulated for the simulated experiments.
